# Pt(II) Rollover Cyclometalated Complexes Supported by 2,2’-Bipyridine *N*‑Oxide: Synthesis, Characterization, and Biological Evaluation

**DOI:** 10.1021/acs.organomet.6c00114

**Published:** 2026-07-14

**Authors:** Antonio Zucca, Giacomo Senzacqua, Antonio Canu, Fabrizio Ortu, Sergio Stoccoro, Maria I. Pilo, Germano Orrù, Giuseppina Pichiri, Sara Fais, Alessandra Scano

**Affiliations:** † 9312 University of Sassari, Department of Chemical, Physical, Mathematical and Natural Sciences, Via Vienna 2, Sassari I-07100, Italy; ‡ Consorzio Interuniversitario Reattività Chimica e Catalisi (CIRCC), Bari 70126, Italy; § School of Chemistry, 4488 University of Leicester, University Road, Leicester LE1 7RH, U.K.; ∥ Department of Surgical Sciences, 3111 University of Cagliari, Cagliari 09124, Italy; ⊥ Department of Medical Sciences and Public Health, University of Cagliari, Cagliari 09124, Italy

## Abstract

Four platinum­(II) rollover cyclometalated complexes derived from 2,2’-bipyridine *N*-oxide (bpy^NO^), [Pt­(bpy^NO^-H)­(DMSO)­Me] (**1a**), [Pt­(bpy^NO^-H)­(PPh_3_)­Me] (**2a**), [Pt­(bpy^NO^-H)­(DMSO)­Cl] (**3a**), and [Pt­(bpy^NO^-H)­(PPh_3_)­Cl] (**4a**), were synthesized, characterized, and evaluated for their antimicrobial and cytotoxic activities. The electron-poor nature of bpy^NO^ enables rollover C–H bond activation at room temperature. NMR and X-ray analysis (**4a**) revealed distinctive features, including an intramolecular interaction between the H^3′^ hydrogen and the N-oxide oxygen. A correlation between the direct ^195^Pt–^31^P coupling constant and the donating properties of cyclometalated ligands has been found, allowing a useful scale of donor properties. A preliminary screening of antimicrobial activity against *E. coli*, *K. pneumoniae*, *S. aureus* *multidrug resistant (MDR)*, *S. pyogenes*, *P. aeruginosa*, and *C. albicans* demonstrated selective activity. Complex **3a** showed the most promising results against *E. coli* and multidrug-resistant *S. aureus*. Significantly, the complexes also exhibited biofilm inhibitory behavior; notably, complex **1a** showed activity against *K. pneumoniae*. Additionally, preliminary cytotoxicity tests on human tumor (HT29) cells and normal (CCD 841 CoN) cells were performed. Among the investigated compounds, **1a**, **2a**, and **3a** exhibited a significant reduction in tumor cell viability, whereas complex **4a** displayed moderate activity. These results establish 2,2’-bipyridine *N*-oxide as a promising ligand scaffold for developing platinum complexes.

## Graphical abstract

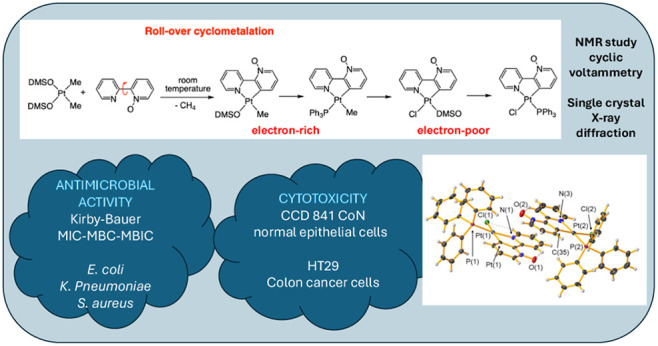

## Introduction

Over recent decades, coordination compounds of transition metals have assumed a key role in several scientific and industrial fields.^1^ Based on the knowledge gained in this area, it is often possible, by adapting the stereoelectronic properties of coordinated ligands, to fine-tune the chemical and physical properties of a complex to target a specific functionality or application. Because of their high tunability, transition-metal complexes are used as advanced materials,^2,3^ in catalysis,^4^ and in medicinal chemistry.^5,6^ In the latter context, metal complexes are currently in clinical development for the treatment of cancer, malaria, and neurodegenerative diseases, while less attention has been paid to their application as antimicrobial agents.^7^ However, this field of application is gaining increasing attention due to the emerging global health threat of antimicrobial resistance (AMR) and multidrug resistance (MDR).

As evidenced by the WHO, due to the use and overuse of antimicrobial drugs over the last century, several classes of microorganisms have evolved, developing resistance to commonly used antimicrobial drugs, so that the return to a preantibiotic era has become a realistic scenario.^8^ Most of the molecules currently under clinical development are merely derivatives of commercial antibiotics, with the consequence that they will likely be rendered ineffective by existing resistance mechanisms.

Transition-metal complexes are extensively investigated for both antimicrobial and anticancer applications. This dual screening approach is often employed to probe how the electronic and structural properties of metal centers can be tuned to interact with distinct biological targets. While the challenge of achieving selectivity between bacterial and cancer cells is significant, understanding the fundamental mechanisms of metal-based cytotoxicity in these different systems is a critical step in the rational design of more specific and potent therapeutic agents.^9^ The rollover cyclometalated Pt­(II) complexes reported here provide a versatile molecular platform to explore these structure–activity relationships, allowing us to modulate reactivity through the careful selection of ancillary ligands.

These mechanistic connections provide significant incentive for exploring metal-based scaffolds for infectious diseases and oncology.

In the search for structurally diverse complexes, cyclometalated complexes of noble metals have emerged as particularly attractive candidates, owing to: (1) the enhanced stability provided by the N^C ligand chelation; (2) their unique catalytic and biocatalytic properties; (3) their versatile interaction profile with biological targets; and finally, (4) the unusually high electronegativity of the metal center, as explained by inverted ligand field theory.^10,11^

The chemistry of cyclometalated complexes of late transition metals is one of the most widely studied and applied in organometallic chemistry.^12^ Besides classical N^C complexes, derived from orthometalation of ligands such as 2-phenylpyridine or benzylimines, variations of the original ligand scaffolds have led to the generation of various new families of cyclometalated complexes, including the recently recognized rollover cyclometallates.^13^

These derivatives are originated through the rollover process, which starts when a chelate complex (Figure 1A) displaces one donor atom (B), allowing an internal rotation that facilitates the activation of a C–H bond initially located far from the metal (C) and permits an agostic interaction (D), leading to the formation of a new metal–carbon bond (E).

**Figure 1:**
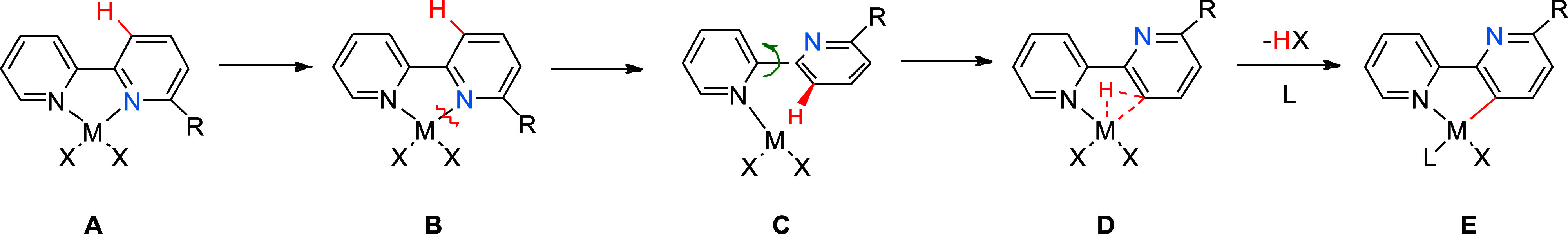
Rollover cyclometalation.

Classical and rollover complexes may be compared in order to highlight both their differences and similarities. Such comparisons are naturally drawn toward the particular role played by the presence of the uncoordinated donor atom, usually a nitrogen, as can be observed in relation to C^N cyclometalated complexes derived from 2-phenylpyridine (phpy) and 2,2’-bipyridine (bpy) (Figure 2). The uncoordinated nitrogen in bipyridine rollover complexes enables interactions or reactions not available to classical cyclometalated species, such as nitrogen protonation, retro-rollover processes, or rollover-catalyzed hydrogen transfer reactions.^14^ Notably, rollover platinum complexes have also exhibited considerable cytotoxic activity.^15–17^

**Figure 2:**
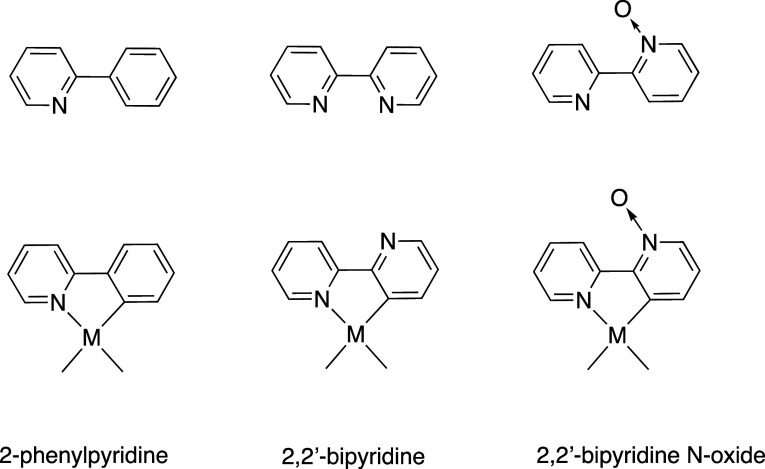
Comparison between three nitrogen ligands and their cyclometalated complexes.

Our group has extensively studied the chemistry of both classical and rollover cyclometalates of late TMs based on the pyridine scaffold,^18–21^ together with their cytotoxic activity and antimicrobial properties.^22–27^ In a continuous effort to analyze variations on this coordination theme, 2,2’-bipyridine N-oxide attracted our attention due to its structural difference compared to the classical bipyridine ligands usually studied. The coordinating behavior of this ligand toward platinum has been the subject of some studies: Puddephatt et al. showed the possibility of obtaining rollover cyclometalated complexes with this ligand, comparing the results with those of the analogous phenanthroline.^28^ Later, Shahsavari, and coworkers thoroughly investigated some aspects of the behavior of platinum-2,2’-bipyridine *N*-oxide complexes, such as oxidative addition reactions, ligand substitution, and kinetic studies, including antitumor activity.^16,29–34^ Apart from the Shahsavari group, to the best of our knowledge, no other studies have been reported on this interesting ligand.

For this reason, we decided to further investigate 2,2’-bipyridine *N*-oxide with the aim of comparing its cyclometalating behavior to that of two comparable and well-known ligands, 2-phenylpyridine and 2,2’-bipyridine (Figure 2), and to conduct a preliminary screening of the antimicrobial and cytotoxic properties of its rollover complexes. This dual strategy is inspired by the idea of reusing existing chemical structures in order to accelerate drug discovery and development.^35^ In particular, the cytotoxic activity was evaluated as an initial assessment of the potential of these compounds against human tumor cell lines, providing a basis for future pharmacological studies aimed at determining their potency and selectivity indices.

In this work, we also describe how these three ligands can lead to the synthesis of their relative cyclometalated complexes, which share many similarities, i.e., giving five-membered planar metallacycles, in part electronically delocalized due to metalloaromaticity.^36^ Nonetheless, a key difference between these complexes stems from the presence of an uncoordinated nitrogen atom in the 2,2’-bipyridine complexes and its subsequent oxidation to an N-oxide in the 2,2’-bipyridine N-oxide complexes, leading to a progressive decrease of donor properties.

## Results and Discussion

### Synthesis and Characterization

The reaction of the electron-rich *cis*-[Pt­(DMSO)_2_Me_2_] complex with 2,2’-bipyridine N-oxide (bpy^NO^) in acetone affords the rollover complex [Pt­(bpy^NO^-H)­(DMSO)­Me], **1a**, in high yields. The rollover C–H bond activation reaction occurs very easily, even at room temperature (Scheme 1), in contrast to 2-phenylpyridine and 2,2’-bipyridine ligands, which require harsh conditions for the C–H bond activation. Such a rapid C–H bond activation at room temperature is not a trivial finding and is an important feature exhibited by the bpy^NO^ ligand. In comparison, rollover cyclometalation of 2,2’-bipyridine only occurs in refluxing toluene.^37^ Complex **1a** may be compared to the analogous dimethyl sulfide complex [Pt­(bpy^NO^-H)­(DMS)­Me] obtained by Puddephatt and coworkers, which was synthesized from the dinuclear complex [Pt_2_Me_4_(μ-DMS)_2_] at room temperature, similarly to **1a**.^28^

**Scheme 1:**
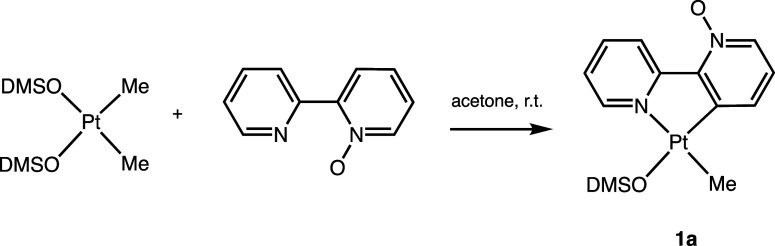
Synthesis of Complex **1a**

Complex **1a** was characterized by elemental analysis and, in solution, by ^1^H NMR spectroscopy, which reveals coordinated methyl and DMSO (δ 0.68 ppm, ^2^J_Pt–H_ = 80.4 Hz; δ 3.29 ppm, ^3^J_Pt–H_ = 18.9 Hz, respectively) in line with the proposed formulation. In the aromatic region, the H^3^ proton is missing and the rollover cyclometalation is confirmed by the signals of the H^4^ and H^5^ protons of the metalated pyridine ring (see Figure 3), which show satellites due to coupling with the ^195^Pt nucleus. In the ^1^H NMR spctrum, two signals are observed significantly deshielded with respect to the free ligand: (1) the signal for H^6’^, at 9.90 ppm (Δδ 1.15 ppm), with satellites due to coupling with the ^195^Pt nucleus (^3^ *J*_Pt–H_ = 17 Hz), confirming coordination of the nitrogen; (2) the H^3′^ proton, whose signal appears at 9.98 ppm (Δδ 1.05 ppm), likely due to cyclometalation, which forces the C_3′_-H and N–O groups into the same plane, in close proximity. This is an important feature of this ligand (*vide infra*), and as a consequence of this interaction, all the protons of the N-coordinated pyridine appear deshielded with respect to the analogous bipyridine complex [Pt­(bpy-H)­(DMSO)­Me], **1b** (Figure 3, e.g.: H^6’^ 9.90 ppm in **1a**, 9.71 ppm in **1b**; H^5′^, 7.44 ppm in **1a** and 7.36 ppm in **1b**). The H^3′^ proton in the bipyridine complex **1b** resonates at 8.29 ppm vs 9.98 ppm in **1a** due to the N–O influence (see Figure 3). H–H 2D COSY and NOESY spectra of **1a** helped in the assignment, showing, in the latter, NOE contacts between coordinated DMSO and methyl hydrogens and the adjacent H^6’^ and H^4^ protons, respectively. The ^13^C NMR spectrum shows all the expected resonances.

**Figure 3:**
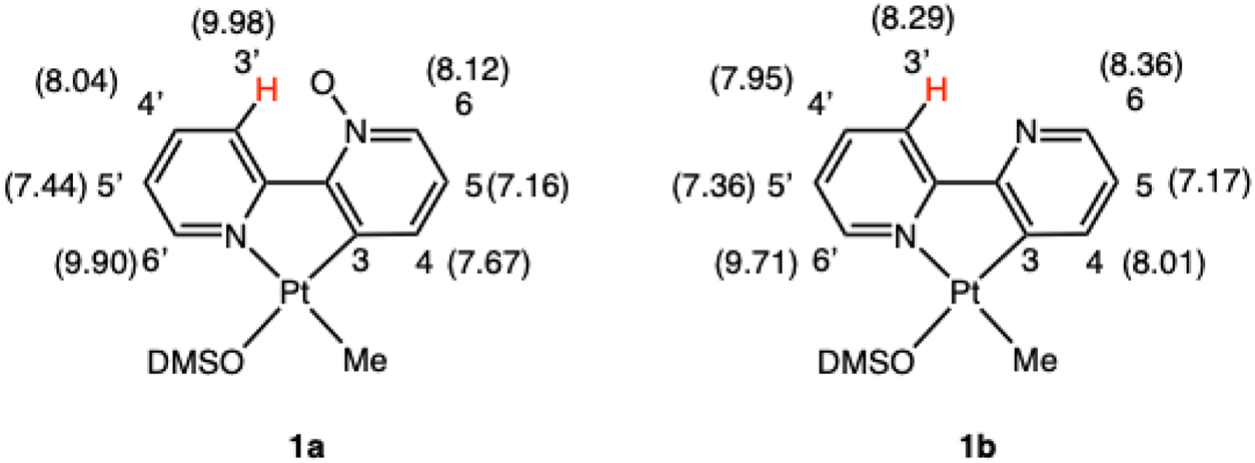
Comparison between complexes **1a** and **1b**, with selected ^1^H NMR data.

From **1a**, the labile DMSO ligand can be readily substituted by PPh_3_ to give complex **2a**, [Pt­(bpy^NO^-H)­(PPh_3_)­Me], previously described by Puddephatt and coworkers,^28^ in high yields.

In order to compare the properties of bpy^NO^ complexes with those of other cyclometalating ligands such as 2-phenylpyridine and 2,2’bipyridine (phpy and bpy), we decided to prepare a series of four complexes with different stereoelectronic properties: two pairs of neutral (PPh_3_ and DMSO) and ionic (methyl and Cl) ligands with different donor properties were chosen for this purpose. The resulting complexes, [Pt­(N^C)­(DMSO)­X] and [Pt­(N^C)­(PPh_3_)­X], **1a**-**4a** (X = Me, Cl; see Figure 4), were compared to other N^C ligands.

**Figure 4:**
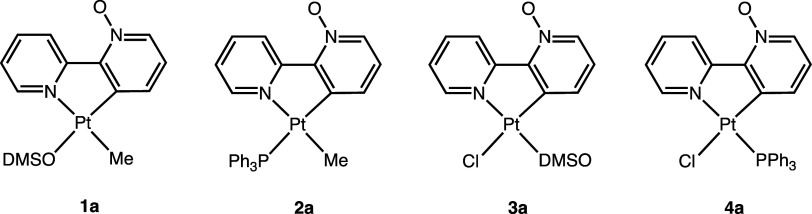
Complexes **1a**–**4a**.

Two geometric isomers potentially exist for these complexes, *i.e., cis-* and *trans*-L–Pt-N isomers (L = DMSO or PPh_3_). However, owing to the large difference in the *trans*-influence of the Cl and Me ligands, only one isomer was observed in all cases: the methyl—i.e., the ligand with the highest *trans*-influence—is always coordinated in *trans* to the nitrogen (**1a** and **2a**), whereas the chloride—i.e., the ligand with the lowest *trans*-influence—is always coordinated in *trans* to the carbon (**3a** and **4a**). These assignments were based on a combination of NMR coupling constant values, NOESY data, and X-ray structural determinations for these and related complexes.

The electron-poor chloride complex [Pt­(bpy^NO^-H)­(DMSO)­Cl], **3a**, was prepared from the reaction of **1a** with aqueous HCl in acetone. Complex **3a** was characterized by means of ^1^H NMR spectroscopy showing the DMSO signal at 3.68 ppm, with a ^3^ *J*_Pt–H_ coupling (23.0 Hz) in line with *trans* S–Pt–N coordination. Also, in this case, the H^6’^ and H^3′^ protons appear strongly deshielded, resonating at 9.80 and 9.86 ppm, respectively. The *trans* S–Pt–N arrangement is demonstrated, *inter alia*, by the Pt–H^6’^ coupling constant (^3^ *J*_Pt–H_ = 40.0 Hz), which is consistent with N–Pt–S coordination. This was lower in **1a** (^3^J_Pt–H_ = 17 Hz) due to the C *trans* influence in the N–Pt–C *trans* coordination. An analogous observation could be made for the H^4^ proton, showing a ^3^ *J*_Pt‑H_ = 50 Hz.

Starting from **3a,** the DMSO ligand may be easily substituted by PPh_3_ to give the corresponding phosphine complex [Pt­(bpy^NO^-H)­(PPh_3_)­Cl], **4a**. The ^1^H and ^31^P NMR spectra of complex **4a** are in agreement with the proposed formulation, in particular, with a P–Pt–N *trans* coordination (^3^ *J*_Pt–P_ = 4191 Hz in **4a** vs 2304 Hz in **2a**). Due to the proximity of the PPh_3_ ligand, the H^4^ and H^5^ protons now appear strongly shielded, resonating at δ 6.60 and 6.42 ppm, respectively. 2D COSY and NOESY spectra further corroborated the proposed assignment.

Complex **4a** was also characterized in the solid state via single-crystal X-ray diffraction (SCXRD). Crystals of complex **4a** suitable for SXCRD studies were obtained by slow diffusion of di-isopropyl ether into an acetone solution of the complex. **4a** crystallizes in the monoclinic space group *P*2_1_/*c*, exhibiting two independent complex molecules in the asymmetric unit. Each complex unit displays a near-regular square planar geometry [Σ_Pt‑X_: Pt(1) 360.4(9)°; Pt(1) 360.0(11)°] with the metal centers lying in close proximity to the coordination plane [Pt(1)···plane 0.048(3) Å; Pt(1)···plane 0.004(4) Å]. Bond distances across the two units are statistically equivalent [e.g., Pt(1)–C(1) 2.003(10) cf. Pt(2)–C(35) 1.998(10) Å] and consistent with previous examples of cyclometalated bipyridyl Pt complexes, including the closely related complex [Pt­(bpy-H)­(Cl)­(PPh_3_)] [Pt–C 2.011(3) Å].^38^ Additional long-range interactions can be observed: (1) the oxygen atoms of the N–O functionalities interact with adjacent hydrogen atoms of the neighboring pyridyl ring [O(1)···H(4) 2.110 Å; O(2)···H(32) 2.114 Å]; (2) the chloride anions show similar long-range interactions with hydrogen atoms of the bipyridyl ligands [Cl(1)···H(1) 2.500 Å; Cl(2)···H(29) 2.525 Å]. The asymmetric unit presents a pseudo-local symmetry consisting of a 2-fold rotation with axis positioned between the two units, parallel to the ligand planes. The two complex molecules of the dimer are held together via π-stacking interactions involving predominantly rings C1–C5 N2 and C29–C33 N3, with distances ranging between 3.638 and 3.910 Å and small tilt angles (range 4.1–4.8°). The π-stacking interactions are also present in the rest of the molecular structure and form a regular 1D stacking of the complex in the lattice (Figure 5).

**Figure 5:**
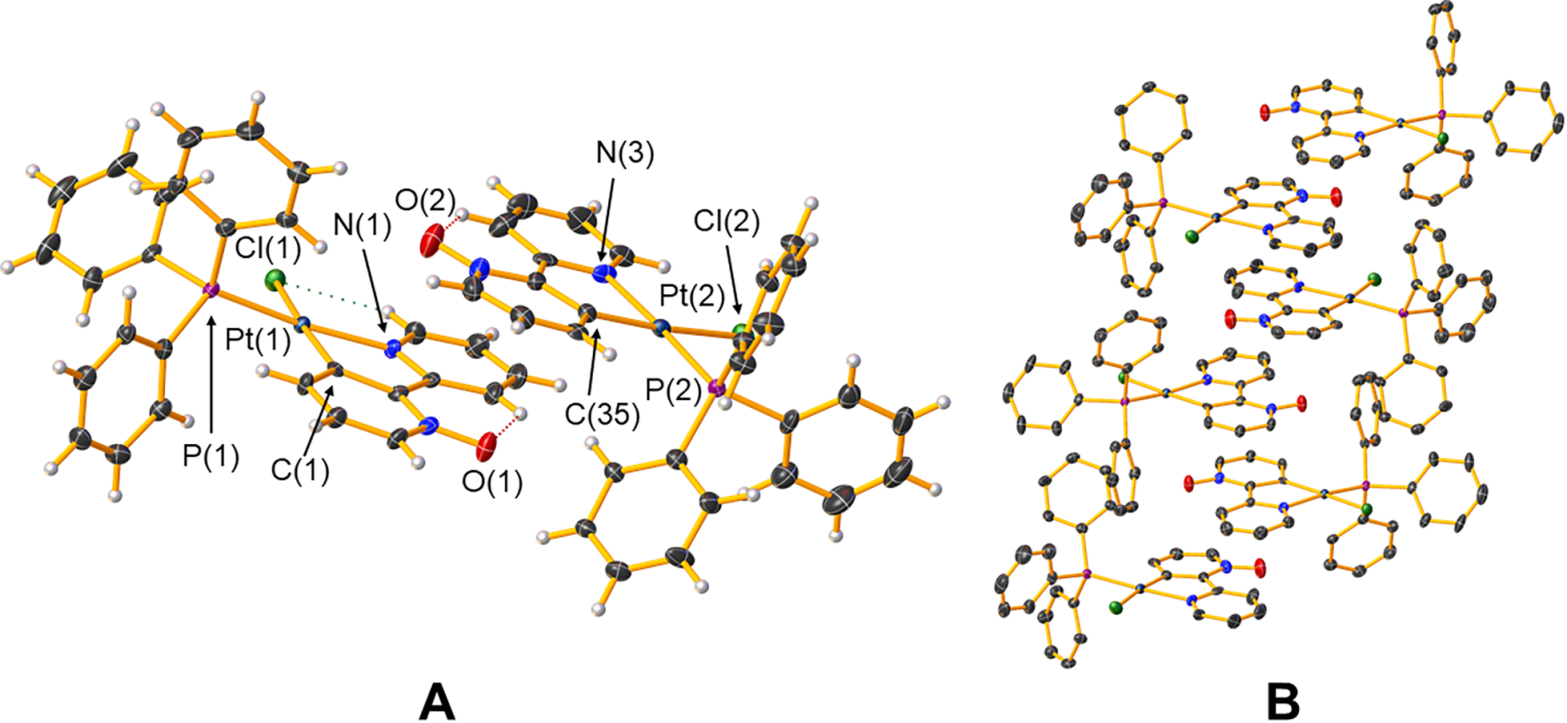
Molecular structure of **4a**: asymmetric unit with selective labeling (**A**) and 1D stacking (**B**). Ellipsoids are shown at 30% probability level; hydrogen atoms in **B** are omitted for clarity.

In order to identify key properties and trends arising from varying stereoelectronic features, we compared the NMR and X-ray data of a series of five-membered N^C Pt­(II) cyclometalated complexes, both classical and rollover, with a range of different donor properties. A comparison of the series of [Pt­(C^N)­(PPh_3_)­Me] complexes (Chart [Fig chart1] and Table S1; see Supporting Information) showed that NMR data may be taken as an indicator of donor properties of the cyclometalated ligands. In particular, whereas ^31^P chemical shifts do not seem to be a good indicator, the direct ^1^ *J*_Pt–P_ coupling constants clearly follow the donor properties of the cyclometalated ligand (Table 1).

**1 chart1:**
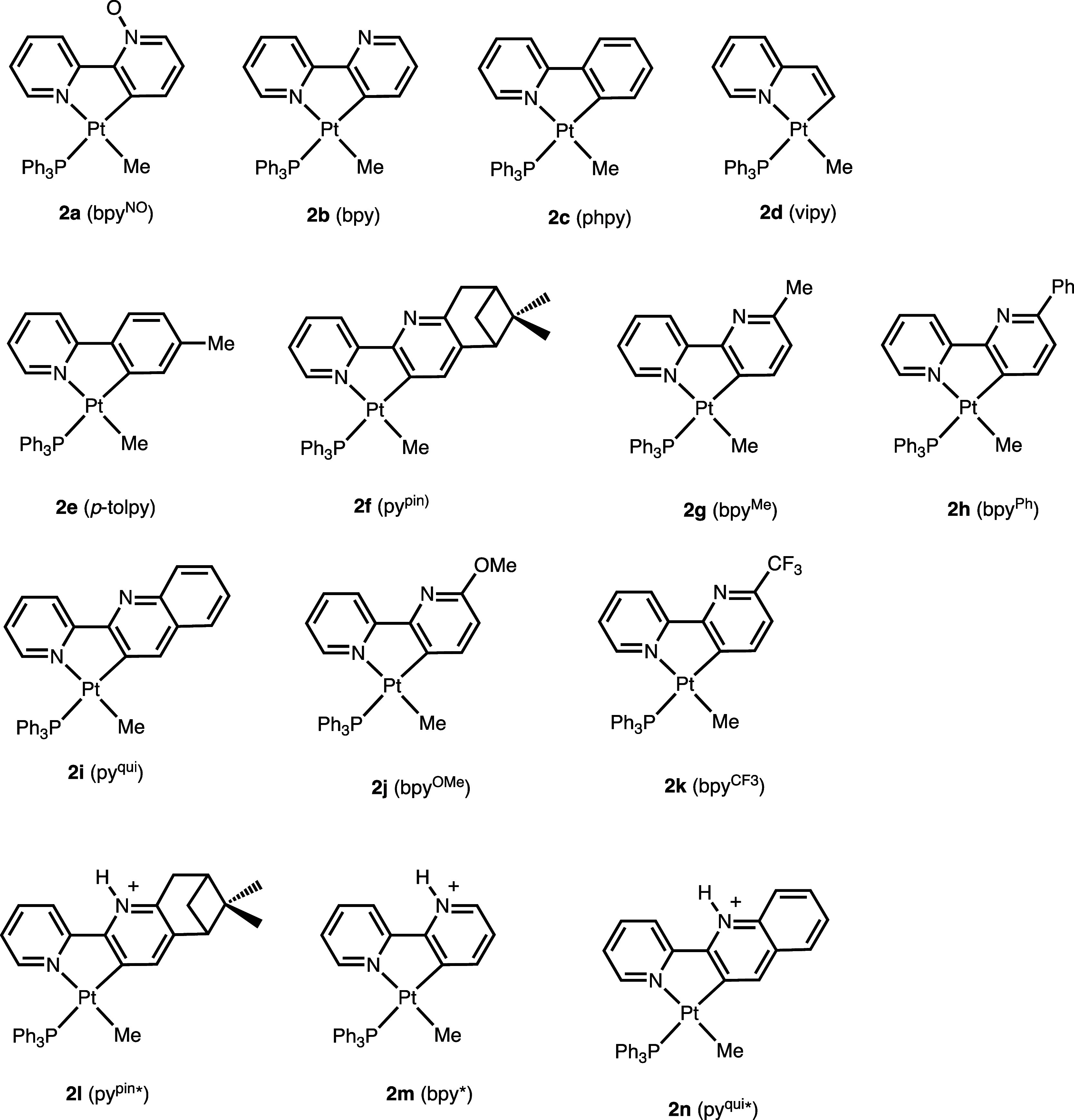
List of neutral [Pt­(N^C)­(PPh_3_)­Me] (**2a**–**h**) and N-protonated [Pt­(N^C*)­(PPh_3_)­Me]^+^ (**2i**–**n**) complexes analyzed in this paper. NMR data are collected in Table 1

**Table 1:** NMR Data for Complexes **2a**–**n** (See Chart [Fig chart1]) (Solvent CDCl_3_)

Complex Pt(N^C)(PPh_3_)Me	N^C ligand	^31^P δ (^1^J_Pt–P_)	^13^C δ (^1^J_Pt–C_)	^1^H δ Me (^3^J_Pt–H_)	References
**2a**	bpy^NO^	32.1 (2304)	150.5 (976)^a^		^28^ ^a^
**2c**	phpy	33.2 (2105)	164.2 (956)^b^	0.79 (83.5)	^39,40^
**2b**	bpy	33.6 (2229)	155.3 (970)		^37^
**2d**	vipy	28.5 (2020)		0.80 (84)	^41^
**2e**	*p*-tol-py	33.1 (2103)		0.69 (83.3)	^42^
**2f**	py^pin^	33.7 (2212)	155.1 (964)	0.72 (83.8)	^43^
**2g**	bpy^Me^	33.6 (2226)		0.73 (83.0)	
**2h**	bpy^Ph^	33.1 (2233)		0.79 (83.0)	^44^
**2i**	pyqui	33.4 (2236)		0.86 (83.1)	^20^
**2j**	bpy^OMe^	35.6 (2257)			^45^
**2k**	bpy^CF3^	32.1 (2279)			^46^
**2l**	py^pin^ *	32.3 (2469)	160.27 (990)	0.78 (82.8)	^43^
**2m**	bpy*	32.1 (2500)	163.4 (997)		^37^
**2n**	pyqui*	32.0 (2507)		0.91 (82)	^20^

a C data, this paper.

b Redetermined. In some cases, J coupling data involving ^195^Pt are difficult to calculate due to broad satellites.

The strongest cyclometalated donor ligand in this series—i.e., the deprotonated 2-vinylpyridine (complex **2d**, [Pt­(vipy-H)­(PPh_3_)­Me])—shows the lowest *J*_Pt–P_ value, 2020 Hz; the magnetic coupling is enhanced further with 2-phenylpyridine (2105 Hz) and with rollover 2,2’-bipyridine (2229 Hz), where N replaces a C–H. Functionalization of the 6’ position on the bipyridine ring affords a range of *J*_Pt–P_ values, which increase according to incremental withdrawing properties of the substituents, i.e., *J*_Pt–P_ values of 2226, 2229, 2233, and 2279 Hz for Me (bpy^Me^), H (bpy), Ph (bpy^Ph^), and CF_3_ (bpy^CF3^) substituents, respectively. In the comparison of ligands bpy^NO^, bpy and phpy (**2a**, **2b**, and **2c**) the trend observed is 2304, 2229, and 2105 Hz, clearly correlating to the presence of NO, N, and CH moieties, respectively. Protonation on the nitrogen of rollover complexes gives the uncommon bpy* ligand, formally a neutral pyridylene,^14^ to give cationic species: **2l**, **2m**, and **2n**. In these cases, very high J_Pt–P_ values were observed (e.g., 2500 Hz for bpy* vs 2229 Hz for bpy-H). The OMe substituent, which showed in the past a dual behavior (electron donor on the π system for mesomeric effect and electron acceptor on the sigma backbone for inductive effects) reveals its withdrawing properties in this analysis, making the electronic properties of the bpy^OMe^ ligand intermediate between those of bpy and bpy^CF3^, as shown by the *J*_Pt–P_ value of 2257 Hz. The overall enhancement of Pt–P coupling constant values is noteworthy, being more than 500 Hz when comparing the cyclometalated complex of 2-vinylpyridine (**2d**, J = 2020 Hz) with the N-protonated complex of 2-pyridylquinoline (**2l**, *J* = 2507 Hz).

In comparison to ^1^ *J*_Pt–P_, the direct Pt–C­(sp^2^) coupling constant of the metalated carbon is difficult to observe, being related to elusive broad satellites of quaternary carbon atoms. When reported, these ^1^ *J*_Pt–C_ couplings follow the same trend as the *J*_Pt–P_ values (*e.g*., 956 Hz for phpy (**2c**), 970 for bpy (**2b**), 976 for bpy^NO^ (**3a**), and 997 Hz for bpy*­(**2m**). The method seems to be particularly sensitive, showing, *inter alia*, clear differences arising from the substitution of a hydrogen (J = 2229 Hz) with a methyl (*J* = 2226 Hz) or phenyl (*J* = 2233 Hz). On the basis of these indications, the C^N-coordinated bpy^NO^ ligand appears to be an extremely electron-poor donor, exhibiting the highest direct *J*(Pt–P) and *J*(Pt–C) values of the neutral complexes in this series. Only the protonated cationic complexes **2l**–**n** show higher values. An interesting observation is that both ^1^ *J*_Pt–P_ and ^1^ *J*_Pt–C_, in mutually *trans* position, vary in the same fashion, i.e., they increase or decrease together. Ultimately, this analysis allowed us to list a trend of donor properties based on cyclometalated N^C ligands (*J*_Pt–P_ in brackets):

vipy (2020) > *p*-tol-py (2013) > phpy (2105) > py^pin^ (2112) > bpyMe (2225) > bpy (2229) > bpy^Ph^ (2233) > pyqui (2036) > bpy^OMe^ (2257) > bpy^CF3^ (2279) > bpy^NO^ (2304) > bpy^Et*^ (2400) > py^pin^* (2469) > bpy* (2500) > pyqui* (2507).

A similar analysis can be made for the [Pt­(N^C)­(DMSO)­Me] complexes (see Table S2). In this case, however, for the DMSO ligand, the ^3^J­(Pt–H) coupling constants are less reliable than direct ^1^J­(Pt–P) values due to the dependence of ^3^J couplings on different parameters, such as bond angles. Data reported in Table S2 for [Pt­(N^C)­(DMSO)­Me] complexes show the same trend for DMSO ^3^J­(Pt–H) values found in [Pt­(N^C)­(PPh_3_)­Me] derivatives. The difference between the values is, however, smaller. ^1^J_Pt–C_ values also show the same trend and may be taken into account for donor trends. Other spectroscopic data of Pt-Me complexes, such as DMSO chemical shifts, appear to be less reliable for simple evaluations.

**Table 2:** Electrochemical Data of Complexes **1a**–4a^a^

Complex	*E*_ox_ (V)
[Pt(bpy^NO^-H)(DMSO)Me] (**1a**)	1.07
[Pt(bpy^NO^-H)(PPh_3_)Me] (**2a**)	0.80
[Pt(bpy^NO^-H)(DMSO)Cl] (**3a**)	–
[Pt(bpy^NO^-H)(PPh_3_)Cl] (**4a**)	0.94

a Potential values (E) reported vs Fc/Fc^+^ redox couple in 0.1 M TEAPF_6_/CH_2_Cl_2_ solvent system.

Less data are available for [Pt­(N^C)­(PPh_3_)­Cl] complexes (Table S3). Here, the phosphorus is coordinated in *trans* to the nitrogen and, hence, in *cis* to the metalated ligand, i.e., the one bearing the substituent’s differences. The trend of ^1^ *J*(_Pt–P_) data is opposite to that of the [Pt­(N^C)­(PPh_3_)­Me] complexes and appears to be very reliable: a very small ^1^ *J*_Pt–P_ data is found for the protonated pyqui* ligand (4143 Hz), and the trend for phpy, bpy, and bpy^NO^ is 4321, 4285, and 4191 Hz, respectively.^47^ Less significant are the ^3^ *J*_Pt–H_ values for coordinated DMSO in [Pt­(N^C)­(DMSO)­Cl] complexes.

In comparison to NMR data, analysis of crystallographic data did not give clear information ( ^48,49^ ); only a few X-ray crystal structures of [Pt­(N^C)­(PPh_3_)­Me] complexes have been determined, containing pyridine N^C cyclometalated ligands (pyridine-N^C-aromatic). A search of the CCDC database (ConQuest, version 2024.2.0 CSD System, searched October 2025) affords only four structures (see Table S3). The small differences in Pt–P bond distances lie within three e.s.d.s (estimated standard deviations) and are therefore not statistically significant. A comparison between NMR and X-ray methods for [Pt­(N^C)­(PPh_3_)­Cl] complexes **4c**, **4b**, and **4a** (N^C = Phpy, bpy, and bpy^NO^ cyclometalated ligands) shows significantly different ^1^ *J*_Pt–P_ values, respectively, of 2105, 2229, and 2304 Hz, with an uncertainty of only a few Hz, whereas Pt–P bond distances do not display significant differences. A similar consideration is valid for [Pt­(N^C)­(DMSO)­Me] complexes.

**Table 3:** Antimicrobial Profile for **1a**–**4a** (0.01 M) with a Set of Gram-Positive and Gram-Negative Bacteria^a^

***Strain control***		*1a*	*2a*	*3a*	*4a*
*E. coli*	*Amp.* 19.6 ± 0.7	–	–	15 mm	–
*S. aureus* *MDR*	*Ox.* 22.6 ± 1.5	14 mm	–	15 mm	15 mm

a Legend: Preliminary antimicrobial activity of complexes **1a**–**4a** is expressed as inhibition zone diameters (mm). The reference antibiotics ampicillin (Amp.) and oxacillin (Ox.) were used as positive controls. “–” denotes no inhibition (diameter <6 mm). Data are reported as mean values of independent measurements.

### Electrochemistry

Cyclic voltammetry characterization of complexes **1a**–**4a** was carried out in methylene chloride solvent, using 0.1 M tetraethylammonium hexafluorophosphate (TEAPF_6_) as supporting electrolyte. The voltammetric responses were recorded at a platinum disk electrode with 100 mV s^–1^ as the potential scan rate.

In the anodic scan, complexes **1a**, **2a**, and **4a** show a broad, irreversible process between 0.80 and 1.07 V (Table 2), whereas no anodic process is detectable in complex **3a**, presumably occurring beyond the solvent system oxidation. The comparison between the two DMSO derivatives (**1a** and **3a**) and between the two PPh_3_ derivatives (**2a** and **4a**) reveals a shift toward more anodic potential values when the -Me ligand is replaced by Cl, due to the higher electron-withdrawing character of chloride. The same feature was previously observed in the analogous Pt­(II) cyclometalated complexes with 2,2’-bipyridines (**2b** and the respective -Cl derivative) and 2-substituted pyridines (**2c** and **2d** and their respective -Cl derivatives).^38,41^ The irreversible behavior of the anodic process in Pt­(II) derivatives is generally ascribed to the instability of the formal Pt­(III) species. However, the involvement of the cyclometalating ligand is usually observed, as well as a lower contribution of the anionic ligand in chloro-derivatives.^38,41^ No cathodic process occurs in the direct scan toward negative potentials.

The voltammetric response of complex **2a** recorded at different potential scan rates (*v*), from 0.010 to 0.500 V s^–1^, showed a linear fit between the increasing current value and *v* ^1/2^, indicative of a charge transfer process under diffusion control. It is interesting to note a change in color of the solution of complex **1a** (where DMSO is the neutral ligand and -Me is the anionic ligand) from yellow to green in the quite short time of the voltammetric experiment. This behavior is probably due to a rearrangement of the complex in solution and can be related to the scarce reproducibility of the voltammetric response.

### Antimicrobial Activity

The group of compounds obtained through Pt­(II) rollover cyclometalation was tested against Gram-positive *Streptococcus pyogenes* *DSM 20565*, a clinically isolated *Staphylococcus aureus* identified as methicillin-resistant and multidrug-resistant *(MRSA/MDR*), Gram-negative *Klebsiella pneumoniae* *DSM 681,* *Escherichia coli* *DSM 1103 and* *Pseudomonas aeruginosa* *DSM 1117*), and yeast *Candida albicans* *DSM 1386*) strains. Remarkably, these represent the first examples of rollover cyclometalated complexes exhibiting antimicrobial and antibiofilm activity (DSM = German Collection of Microorganisms and Cell Cultures GmbH, Braunschweig, Germany). The stock solutions of the compounds were prepared by dissolving them in 1 mL of dimethyl sulfoxide (DMSO) at a concentration of 0.01 M. These stock solutions were then further diluted in the culture medium to achieve final test concentrations.

^1^H NMR spectra of complexes **1a**–**4a** in deuterated DMSO proved their stability in the solvent used for biological tests.

### Agar Diffusion Test

Preliminary antimicrobial screening was performed using the agar diffusion (Kirby–Bauer) method to assess the susceptibility of the tested strains. In this assay, several compounds did not produce measurable inhibition zones (diameter ≈0 mm) against *Klebsiella pneumoniae*, *Streptococcus pyogenes*, *Pseudomonas aeruginosa* *, and* *Candida albicans*. For *Staphylococcus aureus* *MDR* and *Escherichia coli*, varying degrees of inhibition were recorded (Table 3). Specifically, [Pt­(bpy^NO^-H)­(DMSO)­Cl] (**3a**) showed activity against both strains, while [Pt­(bpy^NO^-H)­(PPh_3_)­Cl] (**4a**) and [Pt­(bpy^NO^-H)­(DMSO)­Me] (**1a**) were active against *S. aureus* *MDR*. [Pt­(bpy^NO^-H)­(PPh_3_)­Me] (**2a**) did not display any measurable inhibition zone under the tested conditions. It should be noted that the absence of inhibition zones in the disk diffusion assay does not necessarily imply a lack of intrinsic antimicrobial activity, as the result is strongly influenced by the diffusion rate of the metal complexes within the agar matrix. Therefore, these preliminary observations were further validated using broth microdilution assays (MIC/MBC/MBIC).

### Minimum Inhibitory Concentration (MIC), Minimum Bactericidal Concentration (MBC) Tests, and Anti-Biofilm Assay

#### Antimicrobial Activity: Results

Among the tested Pt­(II) rollover cyclometalated complexes, [Pt­(bpy^NO^-H)­(DMSO)­Cl] (**3a**) exhibited the highest overall antimicrobial and antibiofilm efficacy (Tables 4 and 5).

**Table 4:** Minimum Inhibitory Concentration (MIC), Minimum Bactericidal Concentration (MBC), and Minimum Biofilm Inhibitory Concentration (MBIC) Values of Pt­(II) Rollover Cyclometalated Complexes Bearing Bipyridine *N*-Oxide Ligands against Representative Gram-Negative and Gram-Positive Bacterial Strains

	**[Pt(bpy** ^**NO**^ **-H)(PPh**_**3**_ **)Me]** **2a**			**Pt(bpy** ^**NO**^ **-H)(DMSO)Me]** **1a**		
Strain	MIC (mmol/L)	MBC (mmol/L)	MBIC (mmol/L)	MIC (mmol/L)	MBC (mmol/L)	MBIC (mmol/L)
*E. coli*	2.5	2.5	2.5	2.5	2.5	>10
*K. pneumoniae*	2.5	2.5	>10	2.5	2.5	1.25
*S. aureus* *MDR*	2.5	2.5	2.5	5	5	5

**Table 5:** Minimum Inhibitory Concentration (MIC), Minimum Bactericidal Concentration (MBC), and Minimum Biofilm Inhibitory Concentration (MBIC) Values of Pt­(II) Rollover Cyclometalated Complexes Bearing Bipyridine *N*-Oxide Ligands against Representative Gram-Negative and Gram-Positive Bacterial Strains

	**[Pt(bpy** ^**NO**^ **-H)(DMSO)Cl]** **3a**			**[Pt(bpy** ^**NO**^ **-H)(PPh**_**3**_ **)Cl]** **4a**		
Strain	MIC (mmol/L)	MBC (mmol/L)	MBIC (mmol/L)	MIC (mmol/L)	MBC (mmol/L)	MBIC (mmol/L)
E. coli	1.25	1.25	1.25	>10	>10	>10
K. pneumoniae	>10	>10	>10	>10	>10	>10
S. aureus MDR	1.25	1.25	1.25	10	10	10

This compound showed the lowest MIC, MBC, and MBIC values (1.25 mM) against both *Escherichia coli* and *Staphylococcus aureus* MDR, indicating antibacterial activity. The presence of the DMSO and Cl ligands likely enhances the compound’s solubility and facilitates cellular uptake, contributing to its stronger biological effect. [Pt­(bpy^NO^-H)­(PPh_3_)­Me] (**2a**) also demonstrated moderate antibacterial performance, particularly against *S. aureus* *MDR,* *K. Pneumoniae* *a*nd *E. coli* (MIC = 2.5 mM), although its activity was slightly lower than that of complex **3a**. In contrast, [Pt­(bpy^NO^-H)­(DMSO)­Me] (**1a**) and [Pt­(bpyNO-H)­(PPh_3_)­Cl] (**4a**) showed overall moderate to weak inhibition, with MIC and MBIC values generally above 10 mM for most strains, with the important exception of the MBIC values of **1a** toward *Klebsiella pneumoniae*, the lowest found in this series (1.25 mM).

Regarding antibiofilm activity, it is important to clarify that for complex **3a**, the MBIC values coincide with the MIC values (1.25 mM for both *E. coli* and *S. aureus* *MDR*). This indicates that the antibiofilm effect is observed at concentrations corresponding to the minimum inhibitory concentration rather than at sub-MIC levels. Therefore, the inhibition of biofilm formation by **3a** is likely to be associated with its primary antibacterial activity. In contrast, for complex **1a** against *Klebsiella pneumoniae*, the MBIC value (1.25 mM) was lower than the MIC value (2.5 mM), suggesting that biofilm inhibition may occur even at subinhibitory concentrations. This observation could indicate a partial interference with early biofilm formation processes, such as bacterial adhesion and surface colonization, independent of a complete inhibition of bacterial growth.

The results indicate a nonlinear structure–activity relationship (SAR). The highest antimicrobial activity is observed both for the electron-poor complex **3a**, containing the labile DMSO coligand, and for the electron-rich complex **2a**, bearing the less labile PPh_3_ coligand. In addition, the distinctive activity profile of complex **1a** further highlights the multivariate dependence of the biological response on the nature of the ancillary ligands.

The NMR spectra recorded in deuterated DMSO or DMSO/D_2_O mixtures demonstrate that the cyclometalated ligand is robust and remains chelated under all observed conditions. In complexes **2a** and **4a**, the triphenylphosphine ligand remains coordinated, as evidenced by the persistence of Pt–P coupling constants; similarly, the methyl group in **1a** and **2a** remains firmly bound to the metal center. Conversely, DMSO and Cl act as more labile ligands that may be susceptible to substitution, particularly when positioned *trans* to the cyclometalated carbon, a donor known for its strong *trans* influence. Furthermore, DMSO is a relatively weak σ-donor and exhibits a weak *trans* influence, whereas PPh_3_ is a strong σ-donor and moderate π-acceptor ligand with a pronounced *trans* influence. This distinction can significantly modulate the electron density at the metal center, thereby altering the substitution kinetics and the overall stability of the coordination sphere. Additionally, these electronic factors may influence the bipyridine-*N*-oxide functionality, potentially modulating intermolecular interactions such as hydrogen bonding.

These findings suggest that antimicrobial efficacy arises from a complex interplay between the kinetic lability of the coligands, governed by *trans*-influence and donor/acceptor properties, and the electronic modulation of the cyclometalated scaffold, rather than from a single electronic descriptor. Accordingly, further mechanistic investigations are required to elucidate the molecular basis of these interactions, particularly how substitution kinetics and *trans*-influence effects of the ancillary ligands regulate the accessibility of the platinum center toward biological targets.

Finally, the ability of **3a** to inhibit biofilm formation at low concentrations highlights the potential of this class of Pt­(II) rollover cyclometalated complexes as promising scaffolds for the development of novel antimicrobial and antibiofilm agents.

### Evaluation of the Cytotoxic Effect of Metal Complexes on Human Colon Cell Lines

To evaluate the cytotoxic effect of Pt­(II) rollover cyclometalated complexes, they were tested on two different human colon cell lines: a tumor cell line (HT29) and a normal epithelial cell line (CCD 841 CoN). The cytotoxic profile of Pt­(II) rollover cyclometalated complexes on HT29 colorectal cancer cells is shown in Figure 6.

**Figure 6:**
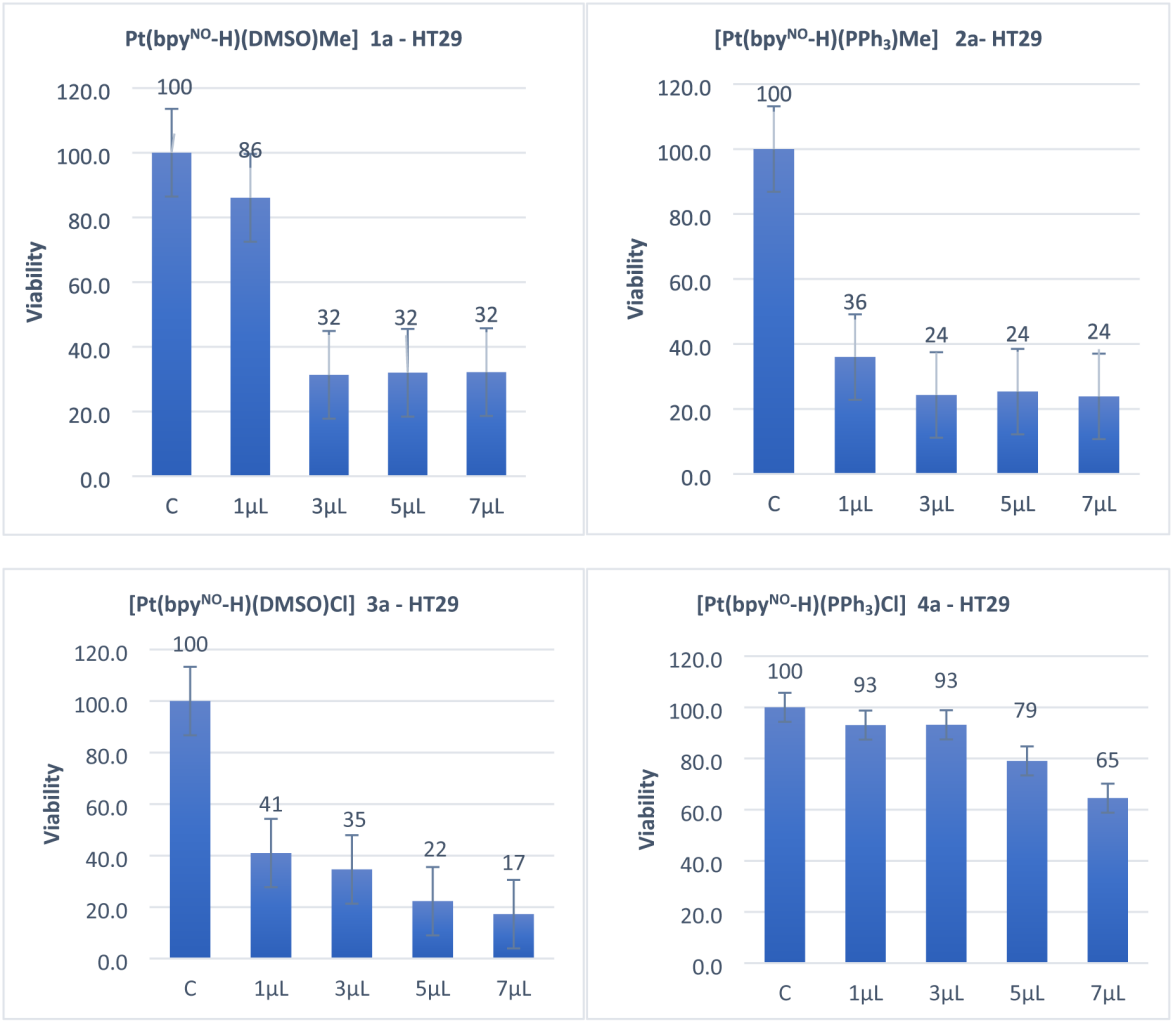
Cell viability of HT29 cells treated with Pt­(II) rollover cyclometalated complexes. Cell viability is expressed as a percentage compared to the untreated control (C), normalized to 100%. Statistical analysis was performed by one-way ANOVA. The overall *p*-value was ≤0.001, with a range between ≤0.0001 and ≤0.008. The *F*-values obtained for each complex are ≤31.5 (**3a**), ≤10.2 (**4a**), ≤288.4 (**2a**), and ≤19.7 (**1a**). The tested volumes (1, 3, 5, and 7 μL) correspond to theoretical final concentrations of 0.1, 0.3, 0.5, and 0.7 mM, respectively, calculated assuming full dissolution in the well.

Data analysis revealed a pronounced cytotoxic effect for all of the tested metal complexes, with a clearly dose-dependent response. The decrease in cell viability observed with an increasing compound concentration suggests a progressive impairment of cellular metabolic activity. Among the investigated compounds, **4a** represented an exception, showing an overall low cytotoxicity across the tested concentration range. A significant reduction in cell viability (64% compared to the untreated control) was detected only at the highest concentration, indicating that this compound may exert milder effects on cell survival compared to the other complexes.

Such behavior could be related to specific structural or coordination features of **4a**, potentially influencing its cellular uptake or interaction with biological targets. Again, complexes **2a** and **3a**, having extreme electronic and coligand properties, appear to be the most active species.

The cytotoxic profile of Pt­(II) rollover cyclometalated complexes on the CCD 841 CoN normal epithelial cell line is shown in Figure 7.

**Figure 7:**
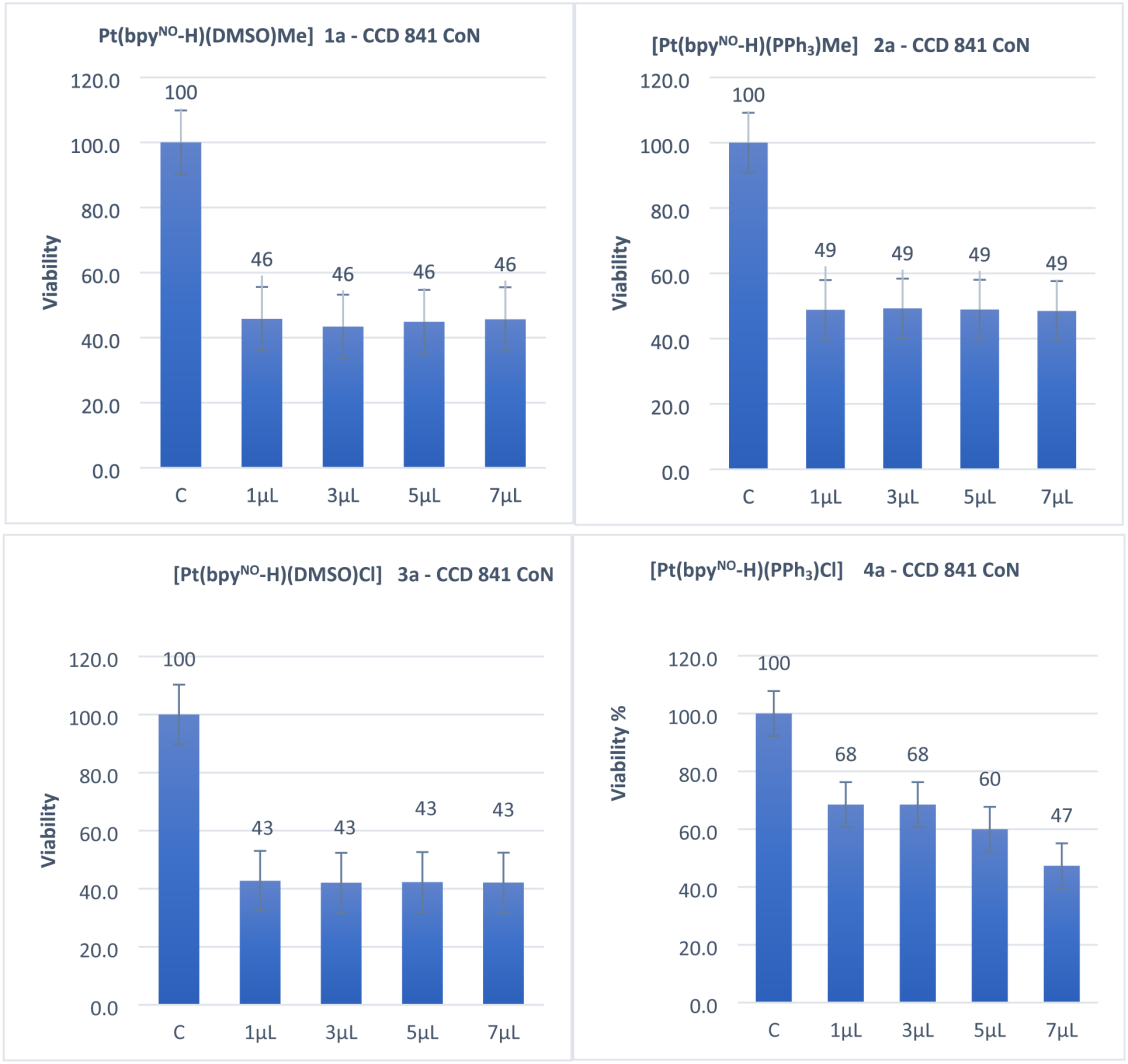
Cell viability of CCD 841 CoN normal epithelial cells treated with Pt­(II) rollover cyclometalated complexes. Cell viability is expressed as a percentage of the untreated control (C), normalized to 100%. Statistical analysis was performed by one-way ANOVA; the overall *p*-value was <0.05 (range: < 0.0001–0.012). The *F* values obtained for each complex are *F* = 63.3 (**3a**), *F* = 65.0 (**4a**), *F* = 113.2 (**2a**), and *F* = 5.0 (**1a**). The tested volumes (1, 3, 5, and 7 μL) correspond to theoretical final concentrations of 0.1, 0.3, 0.5, and 0.7 mM, respectively, calculated assuming full dissolution in the well.

A similar behavior was observed in normal human colon epithelial cells, which showed high cytotoxicity even at the lowest concentrations compared with the control. In this case, however, no clear dose-dependent trend was detected, suggesting that cell viability was markedly affected regardless of the concentration applied. Compound **4a** represented an exception, displaying a less pronounced cytotoxic effect and, unlike the other tested complexes, a distinct dose-dependent response. This finding indicates that **4a** exerts a milder and more predictable influence on cell viability, which could be associated with specific structural or chemical features affecting its biological interactions and cellular uptake.

## Conclusions

In this study, we have reported the synthesis, characterization, and biological activity of a series of Pt­(II) rollover complexes with an electron-poor 2,2’-bipyridine ligand, 2,2’-bipyridine N-oxide (bpy^NO^). Rollover cyclometalation is a unique coordinating behavior, which has a distinctive feature in having a free donor atom, able to furnish intermolecular interactions not available to classical cyclometalated complexes. In this case, the uncoordinated nitrogen is oxidized, and the presence of the N–O functionality enables intra- and intermolecular interactions not available to other bipyridines.

This research demonstrates that 2,2’-bipyridine N-oxide represents an advancement in the development of electron-poor ligands for platinum­(II) rollover cyclometalation. The electron-withdrawing nature of the N-oxide functionality considerably enhances the reactivity of bpy^NO^ toward C–H bond activation, enabling rollover cyclometalation to proceed under mild conditions, even at room temperature, compared to the harsh conditions required for the parent 2,2’-bipyridine ligand.

The four complexes [Pt­(bpy^NO^-H)­(L)­X] (where L = DMSO, PPh_3_; X = Me, Cl) studied in this work exhibit distinctive structural features. The rollover cyclometalation imposes a planar arrangement that brings the H^3′^ proton into close proximity with the N–O functionality, contributing in a unique way to the overall behavior of these rollover complexes. This structural constraint results in characteristic NMR signatures, particularly the significant deshielding of H^3′^ (e.g., δ 9.98 ppm in **1a** vs 8.29 ppm in the analogous bipyridine complex **1b**). The H^3′^···O interaction is confirmed by the X-ray structure of complex **4a**, which shows 2.110 and 2.114 Å O···H distances.

The direct ^195^Pt–^31^P coupling constant observed in NMR spectra correlates notably with the donating ability of cyclometalated ligands, thereby providing a practical scale for evaluating donor properties across an extensive series of cyclometalated ligands, including phenyl- and vinyl-pyridines. This represents a result of considerable significance, as it proves highly sensitive to even subtle differences in donor capacity.

Furthermore, the complexes show selective antimicrobial activity against clinically relevant pathogens, with effectiveness against Gram-negative bacteria (*E. coli*, *K. pneumoniae*) and Gram-positive bacteria (*S. aureus* *MDR).* Beyond planktonic bacterial inhibition, the complexes show biofilm inhibitory properties, which are crucial for treating persistent infections. Complexes **2a** and **3a**, [Pt­(bpy^NO^-H)­(PPh_3_)­Me] and [Pt­(bpy^NO^-H)­(DMSO)­Cl], emerged as the most active compounds, suggesting a complex structure–activity relationship. Interestingly, these results may be connected to electrochemical data: among the series of complexes **1a**-**4a**, complex **2a** is the most electron-rich, showing the lowest potential (0.80 V), and **3a** is the most electron-poor, with a higher, outside-scale potential. Complexes **1a** and **4a** lie in the middle, with potentials of 1.07 and 0.94 V, respectively. This suggests that the most easily oxidizable complex (**2a**) and the hardest to be oxidized (**3a**) have higher antimicrobial activity, whereas the intermediate complexes **1a** and **4a** show worse results, with the important exception of the antibiofilm activity of **1a** against *Klebsiella pneumoniae*.

Indeed, the antibiofilm activity found for complex **1a** against *K. pneumoniae* is particularly noteworthy. While the MBIC value of 1.25 mmol/L (corresponding to 599 μg/mL) may be low in absolute terms, it remains of interest. Such interest stems from the aggressive nature of this Gram-negative bacterium and the notorious difficulty of targeting it when organized in biofilms.

Additionally, the study included a preliminary cytotoxicity screening on human cell lines of complexes **1a**–**4a**, showing that complexes **1a**, **2a**, and **3a** exhibited significant tumor cell toxicity against human tumor (HT29) cells. The robust N^C chelation ensures stability under physiological conditions, while the synthetic versatility of the scaffold allows for further optimization. Although direct comparisons with clinically used platinum drugs such as cisplatin, carboplatin, and oxaliplatin were beyond the scope of the present study, some relevant differences can already be highlighted. Classical platinum-based drugs mainly exert their anticancer activity through DNA cross-linking, whereas the cyclometalated Pt­(II) complexes investigated here possess distinct structural and electronic features that may promote alternative or multitarget mechanisms of action. In particular, complexes **1a** and **3a** exhibited promising antiproliferative activity, together with preliminary selectivity toward HT29 cells over CCD 841 CoN cells, as well as antimicrobial and antibiofilm properties. These findings suggest a biological profile that differs from conventional platinum chemotherapeutics and supports further investigations aimed at direct head-to-head comparisons with standard platinum drugs through standardized IC50, selectivity, and mechanistic studies.

This work establishes 2,2′-bipyridine *N*-oxide as a powerful tool in the development of new metallodrugs to combat antimicrobial resistance (AMR). To summarize, 2,2’-bipyridine *N*-oxide has proven to be a noteworthy electron-poor ligand that facilitates platinum­(II) rollover cyclometalation, leading to complexes with interesting antimicrobial potential. To the best of our knowledge, these are the first rollover complexes with antimicrobial activity reported to date. This work opens new avenues for developing metallodrugs to combat the growing threat of antimicrobial resistance and establishes a basis for future research in this critical area of medicinal chemistry.

## Experimental Section

All of the solvents were purified and dried according to standard methods. ^1^H, ^13^C, and ^31^P NMR spectra were recorded with a Bruker Avance III 400 spectrometer. Chemical shifts are given in ppm relative to internal TMS for ^1^H and ^13^C and external 85% H_3_PO_4_ for ^31^P; *J* values are given in Hz (see Chart [Fig chart2] for the numbering scheme used). Two-dimensional spectra were obtained by using standard pulse sequences. All of the reactions were conducted under an argon atmosphere.

**2 chart2:**
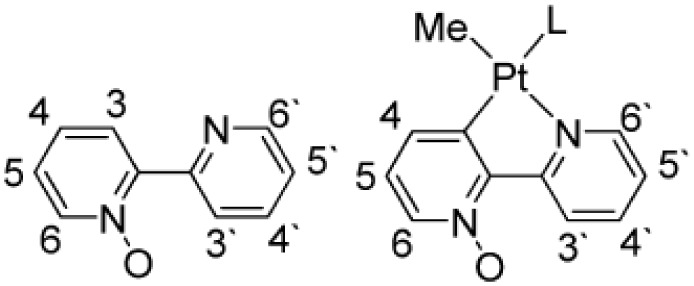
NMR Labeling Scheme

Cyclic voltammetry (CV) characterizations were performed in a three-electrode, single-compartment cell under an argon atmosphere. A Pt disk (2 mm diameter) was the working electrode, Ag/AgCl with a suitable salt bridge was the reference electrode, and a Pt wire was the counter electrode. The working electrode was polished with 1 and 0.3 μm alumina powder, then rinsed with distilled water and acetone before each experiment. Methylene chloride (anhydrous, ≥99.8%, packaged under nitrogen) was used as the solvent, containing 0.1 M tetraethylammonium hexafluorophosphate (TEAPF_6_) as the supporting electrolyte. All experiments were performed using an AUTOLAB PGSTAT12 instrument interfaced with a personal computer equipped with software NOVA 2.1. All potential values are finally referred to the half-wave potential of the ferrocene/ferricenium ion (Fc/Fc^+^) redox couple, as measured in CV tests in 0.1 M TEAPF_6_/CH_2_Cl_2_ solvent system.

### Agar Diffusion Test

The antimicrobial activity of the tested compounds was evaluated according to the Clinical and Laboratory Standards Institute (CLSI) guidelines. Preliminary screening was performed using the agar diffusion (Kirby–Bauer) method, which allows a rapid assessment of bacterial susceptibility or resistance to the tested formulations. Briefly, each strain was inoculated onto the surface of the plate using a sterile buffer with a standardized inoculum of 5 × 10^7^ CFU for bacteria and 5 × 10^6^ CFU for yeast. Fifty microliters of each substance were applied in triplicate to each microbial strain. Petri dishes were incubated at 37 °C for 24 h. After incubation, the diameter of the inhibition zone was measured in millimeters.

### Minimum Inhibitory Concentration (MIC) and Minimum Bactericidal Concentration (MBC) Tests

The antimicrobial activity of the compounds was further evaluated using the standard broth dilution method in sterile 96-well microplates. Serial dilutions of each compound, starting at 0.01 M in nutrient broth, were tested for each strain with a final inoculum of 1 × 10^6^ CFU/mL. Experiments were performed in triplicate. After 24–48 h of incubation at 37 °C under appropriate conditions, the minimum inhibitory concentration (MIC) was determined as the lowest compound concentration preventing visible growth, measured at 550 nm using a Multiskan FC microplate photometer (Thermo Fisher Scientific IT, Milan, Italy). To determine the minimum bactericidal concentration (MBC), 50 μL aliquots from wells showing no growth were plated onto Mueller–Hinton agar and incubated for 24  h at 37 °C. The MBC was defined as the lowest concentration capable of killing ≥99.9% of the bacterial population.

### Anti-Biofilm Assay

The minimum biofilm inhibitory concentration (MBIC) was determined using a modified crystal violet staining protocol based on the Montana University Center for Biofilm Engineering (http://www.biofilm.montana.edu, accessed on 31 March 2025). After 48 h of incubation, the medium was removed, and wells were gently washed three times with 0.9% NaCl. Then, 100 μL of 0.1% crystal violet solution was added to each well for 10 min. Excess dye was discarded, wells were washed three times with 0.9% NaCl, and the bound dye was solubilized with 200 μL of 30% acetic acid. Biofilm formation was quantified by measuring absorbance at 620 nm using a Multiskan FC microplate photometer (Thermo Fisher Scientific IT, Milan, Italy).

### Cytotoxic Effect

Human colorectal adenocarcinoma cells HT29 (ATCC HTB-38), obtained from the National Institute for Cancer Research–Advanced Biotechnology Center (ICLC, Genoa, Italy), were cultured in RPMI 1640 medium supplemented with 10% fetal bovine serum (FBS), 100 U/mL penicillin, 100 μg/mL streptomycin, 2 mM l-glutamine, and 1% nonessential amino acids. Confluent cells were detached by treatment with trypsin/EDTA and subsequently seeded at a density of 1.5 × 10^4^ cells/cm^2^. Normal human colon epithelial cells CCD 841 CoN (ATCC CRL-1790) were cultured in Eagle’s Minimum Essential Medium (EMEM) supplemented with 10% (v/v) FBS, 100 U/mL penicillin, and 100 μg/mL streptomycin. Cell cultures were maintained at 37 °C in a humidified atmosphere containing 5% CO_2_ and subcultured every 2–3 days. The metal complexes were dissolved to obtain stock solutions at a final concentration of 0.01 M. After cell adhesion and stabilization, aliquots of 1 μL, 3 μL, 5 μL, and 7 μL of these 10^–2^ M solutions were added to the culture medium (final volume = 100 μL), corresponding to final concentrations of 0.1, 0.3, 0.5, and 0.7 mM, respectively, in both cell lines. Cells were then incubated under standard conditions (37 °C, 5% CO_2_) for 24 h. Untreated cells were used as negative controls. At the end of the exposure period, cell viability was determined using the MTT assay with the Cell Proliferation Kit I (MTT) (Roche Diagnostics, code 11465007001), according to the manufacturer’s instructions. Results were expressed as the percentage of cell viability relative to untreated control cells, which were considered 100% viable.

### Crystallography

#### Crystallographic Method

The crystal data for compound **4a** are compiled in Table S1 (Supporting Information). Crystals were examined using a Bruker D8 Quest diffractometer with a Photon III detector and a microfocus source with Cu–Kα radiation (λ = 1.54178). Intensities were integrated from data recorded on 1.0° frames by ω rotation. A multiscan method absorption correction with a beam profile was applied.^50,51^ The structures were solved using SHELXS or SHELXT;^52^ the data sets were refined by full-matrix least-squares on reflections with *F* ^2^ ≥ 2σ­(*F* ^2^) values, with anisotropic displacement parameters for all non-hydrogen atoms, and with constrained riding hydrogen geometries;^53^ *U*_iso_(H) was set at 1.2 (1.5 for methyl groups) times *U*_eq_ of the parent atom. The largest features in final difference syntheses were close to heavy atoms and were of no chemical significance. SHELX^52,53^ was employed through OLEX2 for structure solution and refinement.^54^ The structure has been deposited with the Cambridge Crystallographic Data Centre (CCDC 2512771). This information can be obtained free of charge from www.ccdc.cam.ac.uk/data_request/cif.

## Preparations

### **1a**, [Pt­(bpy^NO^-H)­(DMSO)­Me]

To a solution of [Pt­(DMSO)_2_Me_2_] (221.5 mg; 0.581 mmol) in acetone (20 mL), an equimolar amount of bpy^NO^ was added under stirring. The reaction was heated to 60 °C for 1 h, then the solution was concentrated to a small volume and diethyl ether was added. The precipitate formed was filtered under vacuum, washed with diethyl ether, and dried to give the analytical sample with a 95% yield. The reaction also occurs at room temperature, requiring longer reaction times. M.P. = 210 °C. Anal. Calc for C_13_H_16_N_2_O_2_PtS: C, 33.99; H, 3.51; N, 6.10; found C, 33.84; H, 3.86; N, 6.21. ^1^H NMR (CDCl_3_): δ­(ppm)= 9.98 (d; 1H; *J*_H_-_H_= 8.5 Hz, H^3′^); 9.90 (dd with sat, *J*_Pt–H_ = 17 Hz, J_H‑_. _H_ = 5.6, 1.8, 0.8 Hz, 1H, H^6’^); 8.12 (dd, *J*_H–H_ = 0.9; 6.5 Hz, 1H, H^6^); 8.04 (H^4’^); 7.67 (*J*_Pt–H_ = 63 Hz, 1H, H^4^); 7.44 (H^5′^); 7.16 (dd, *J*_Pt–H_ = 23 Hz, *J*_H–H_ = 6.6; 7.4 Hz, 1H, H^5^); 3.29 (s with sat, *J*_Pt–H_ = 18.9 Hz, 6H, Me (DMSO)); 0.68 (s with sat, *J*_Pt–H_ = 80.4 Hz, 3H, Me). ^13^C NMR (CDCl_3_): δ­(ppm)= 157.0 (C^2^ or C^2’^), 153.3 (C^3^), 150.9 (C^6’^), 149.3 (C^2^ or C^2^), 138.9 (C^4’^), 137.8 (C^6^), 130.1 (C^4^), 126.2 (C^3′^), 125.3 (C^5′^), 123.7 (C^5^), 43.9 (DMSO, *J*_Pt–C_ = 42.8 Hz), (CH_3_, *J*_Pt–C_ = 761 Hz).

### **2a**, [Pt­(bpy^NO^-H)­(PPh_3_)­Me]

To a solution of **1a** (60 mg; 0.13 mmol) in acetone (29 mL), an equimolar amount of PPh_3_ was added under stirring. The solution was stirred at room temperature for 2 h, then it was concentrated to a small volume and treated with diethyl ether to give a precipitate, which was filtered under vacuum and dried to give the analytical sample. Yield 75%. ^31^P NMR (CDCl_3_): δ (ppm)= 32.11­(s with sat, *J*_Pt–P_ = 2304 Hz).

### **3a**, [Pt­(bpy^NO^-H)­(DMSO)­Cl]

To a solution of **1a** (70 mg; 0.15 mml) in acetone (10 mL), 1.6 mL of 0.1 M aqueous HCl was added. The mixture was stirred at room temperature for 30 min, during which a precipitate formed. The precipitate was filtered under vacuum and dried to give the analytical sample with a 75% yield. M.P. > 260 °C. Anal. Calc for C_12_H_13_ClN_2_O_2_PtS: C, 30.04; H, 2.73; N, 5.84; found C, 30.37; H, 2.88; N, 5,93. ^1^H NMR (CDCl_3_): δ­(ppm)= 9.86 (dd, 1H, *J*_H–H_ = 8.4, 1.3 Hz, H^3′^); 9.80 (dd with sat, 1H, *J*_Pt–H_ = 40.0 Hz, *J*_H–H_ = 5.8, 1.5 Hz, H6’); 8.40 (dd with sat, 1H, *J*_Pt–H_ = 50.0 Hz, *J*_H–H_ = 8.0, 0.9 Hz, H^4^); 8.12 (d, 1H, *J*_H–H_ = 6.5, 1.0 Hz, H^6^); 8.08 (m, 1H, *J*_H–H_ = 8.4, 7.5, 1.7 Hz, H^4’^); 7.49 (ddd, 1H, *J*_H–H_ = 7.5, 5.8, 1.6 Hz, H^5′^); 7.08 (dd, 1H, *J*_H–H_ = 8.0, 6.5 Hz, H^5^); 3.68 (s with sat, *J*_Pt–H_ = 23.0 Hz, 6H, Me (DMSO)). ^13^C NMR (CDCl_3_): δ­(ppm)= 150.4 (C^6’^), 141.5 (C^4’^), 137.6 (C^6^), 131.2 (C^4^), 126.2 (C^3′^), 124.5 (C^5′^), 124.4 (C^5^), 47.1 (DMSO).

### **4a**, [Pt­(bpy^NO^-H)­(PPh_3_)­Cl]

To a solution of **1a** (70 mg; 0.15 mmol) in acetone (10 mL), 1.6 mL of 0.1 M aqueous HCl was added. The mixture was stirred at room temperature for 1.5 h, then PPh_3_ was added (43.85 mg; 0.17 mmol), and the mixture was stirred for another hour. At the end, the solution was concentrated to a small volume and treated with diethyl ether to give a pale yellow precipitate, which was filtered under vacuum and dried to give the analytical sample as a pale yellow solid. Yield 95%. M.P. > 250 °C. ^1^H NMR (CDCl_3_): δ­(ppm) = 10.6 (m with sat,1H, *J*_Pt–H_ = ca. 28 Hz, H^6’^); 9.90 (d, 1H, *J*_H–H_ = 6.3 Hz, H^3′^); 8.08 (m, 1H, *J*_H–H_ = 1.2; 8.0 Hz, H^6^); 7.95 (d, 1H); 7.80 (m, 6H); 7.56–7.38 (m, 9H); 7.52 (multiplet overlapping, 1H, H^4’^); 6.60 (dd with sat, *J*_Pt–H_ = 58 Hz, 1H, *J*_H–H_ = 2.2; 7.8 Hz, H^4^); 6.40 (m, 1H, *J*_H–H_ = 7.8, 6.8 Hz, H^5^).^31^P NMR (CDCl_3_): δ­(ppm)= 21.89 (s with sat, *J*_Pt–P_ = 4191 Hz). ^13^C NMR (CDCl_3_): δ­(ppm) 149.5 (C^6^), 140.4, 136.0, 135.4 (d with sat, *J*_P–C_ = 11.2 Hz, *J*_Pt–C_ = 28.5 Hz, H*ortho* PPh_3_), 131.2 (s, C*para* PPh_3_), 129.6 (d, *J*_P–C_ = 64.2 Hz, C*ipso* PPh_3_), 128.2 (d, *J*_P–C_ = 11.5 Hz, C*meta* PPh_3_), 126.1, 124.7, 124.7, 123.9.

## Supplementary Material



## Notes

NMR spectra of complex **1a**; Figures S7–S8: NMR spectra of complex **2a**; Figures S9–S14: NMR spectra of complex **3a;** Figures S15–S17: NMR spectra of complex **4a**. **Table S1**: selected crystallographic data for complex **4a**. **Table S2**: NMR data for other complexes. **Table S3**: comparison between Pt–P and Pt–S bond distances in complexes [Pt­(N^C)­(PPh_3_)­Me] and [Pt­(N^C)­(DMSO)­Me] (PDF)
