# Supplementary material for: Pt(II) Rollover Cyclometalated Complexes Supported by 2,2’-Bipyridine N‑Oxide: Synthesis, Characterization, and Biological Evaluation
Source: Organometallics. 2026 Jul 14;45(14):1698–711. doi: 10.1021/acs.organomet.6c00114 (PMC13418185; doi:10.1021/acs.organomet.6c00114)
Supplement: Supplementary file 1 [file om6c00114_si_001.pdf]

Supplementary information

Pt(II) rollover cyclometalated complexes supported by 2,2'-bipyridine N-oxide: synthesis, characterisation and biological evaluation

Antonio Zucca,[a,b]* Giacomo Senzacqua,[a,b] Antonio Canu,[a] Fabrizio Ortu,[c] Sergio Stoccoro,[a,b] Maria I. Pilo,[a] Germano Orrù,[d] Giuseppina Pichiri,[e] Sara Fais[d] and Alessandra Scano[d]

a) University of Sassari, Department of Chemical, Physical, Mathematical and Natural Sciences, Via Vienna 2, I-07100 Sassari, Italy
b) Consorzio Interuniversitario Reattività Chimica e Catalisi (CIRCC) Bari, Italy
c) School of Chemistry, University of Leicester, University Road. Leicester, LE1 7RH (UK)
d) Department of Surgical Sciences, University of Cagliari, Cagliari 09124, Italy
e) Department of Medical Sciences and Public Health, University of Cagliari, 09124, Cagliari, Italy

Email: zucca@uniss.it (AZ); alessandra.scano77@unica.it (AS)

Table of Contents

**Pages 2-5**	Figures S1-S6: NMR spectra of complex **1a**
**Page 6**	Figures S7-S8: NMR spectra of complex **2a**
**Pages 7-9**	Figures S9-S14: NMR spectra of complex **3a**
**Pages 10-12**	Figures S15-S20: NMR spectra of complex **4a**
**Page 13**	**Table S1**: Selected crystallographic data for complex **4a**
**Page 14**	**Table S2**: NMR data for other complexes
**Page 14**	**Table S3**: Comparison between Pt-P and Pt-S bond distances in complexes [Pt(N^C)(PPh_3)Me] and [Pt(N^C)(DMSO)Me]

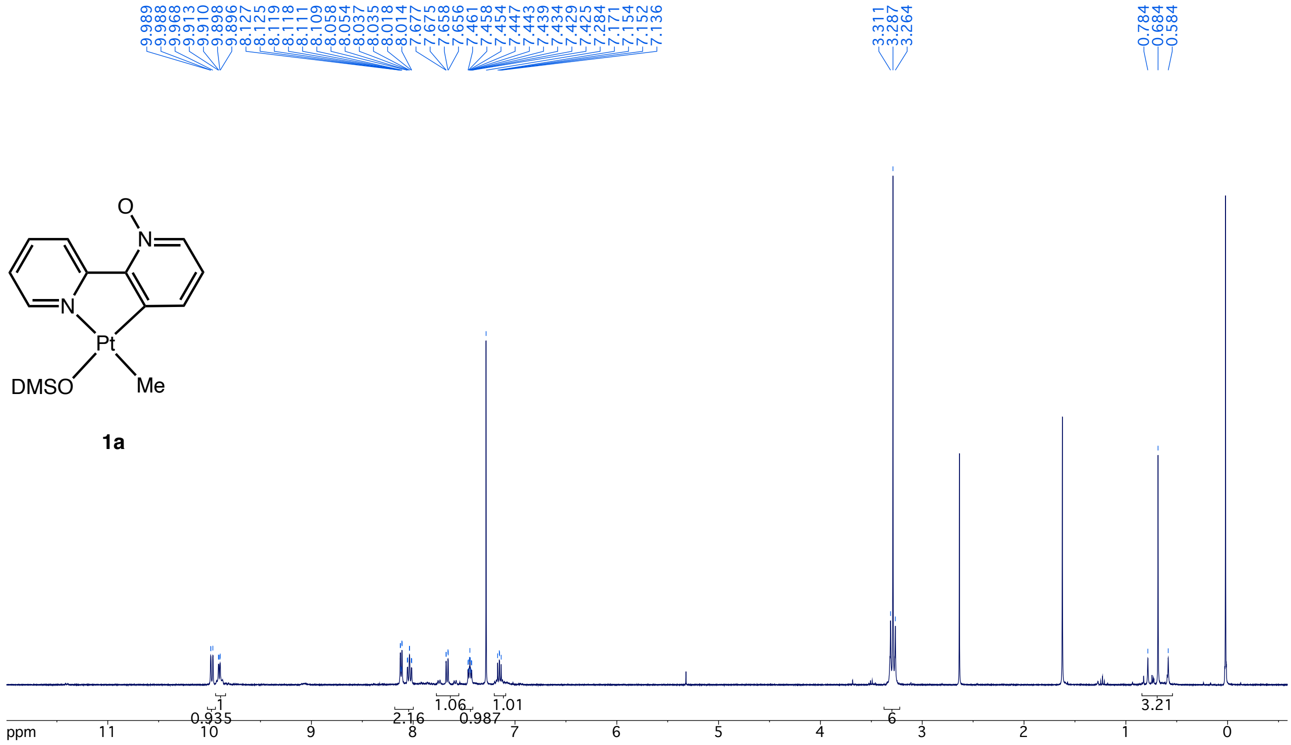

Figure S1. ^{1}H NMR spectrum ($CDCl_3$) of **1a**

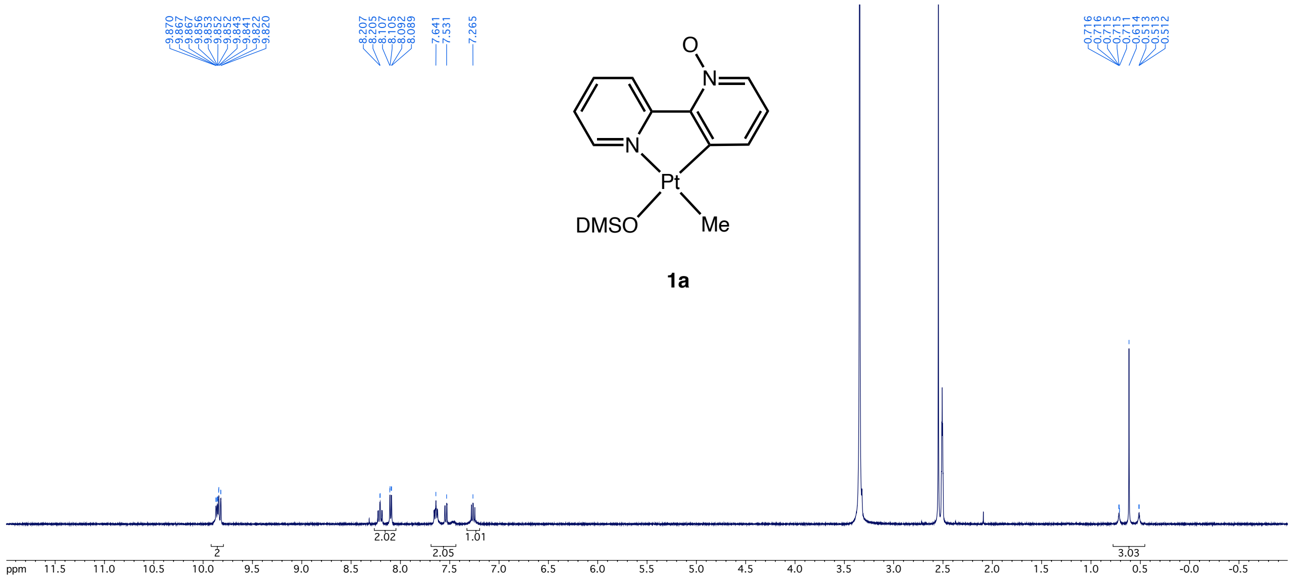

Figure S2. ^{1}H NMR spectrum (DMSO) of **1a**

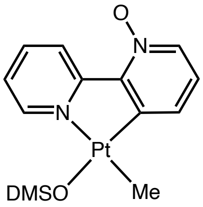

1a

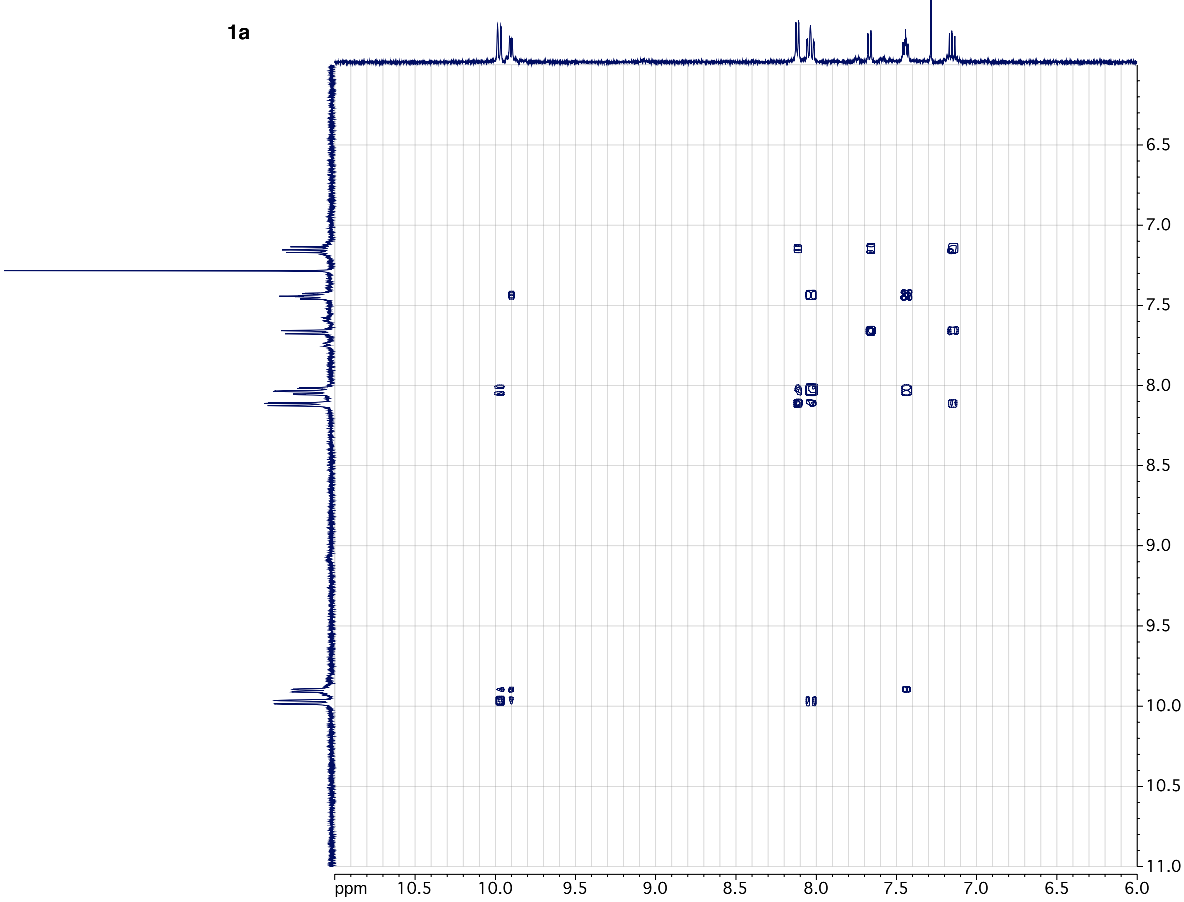

Figure S3. H-H COSY NMR spectrum ($CDCl_3$) of **1a**

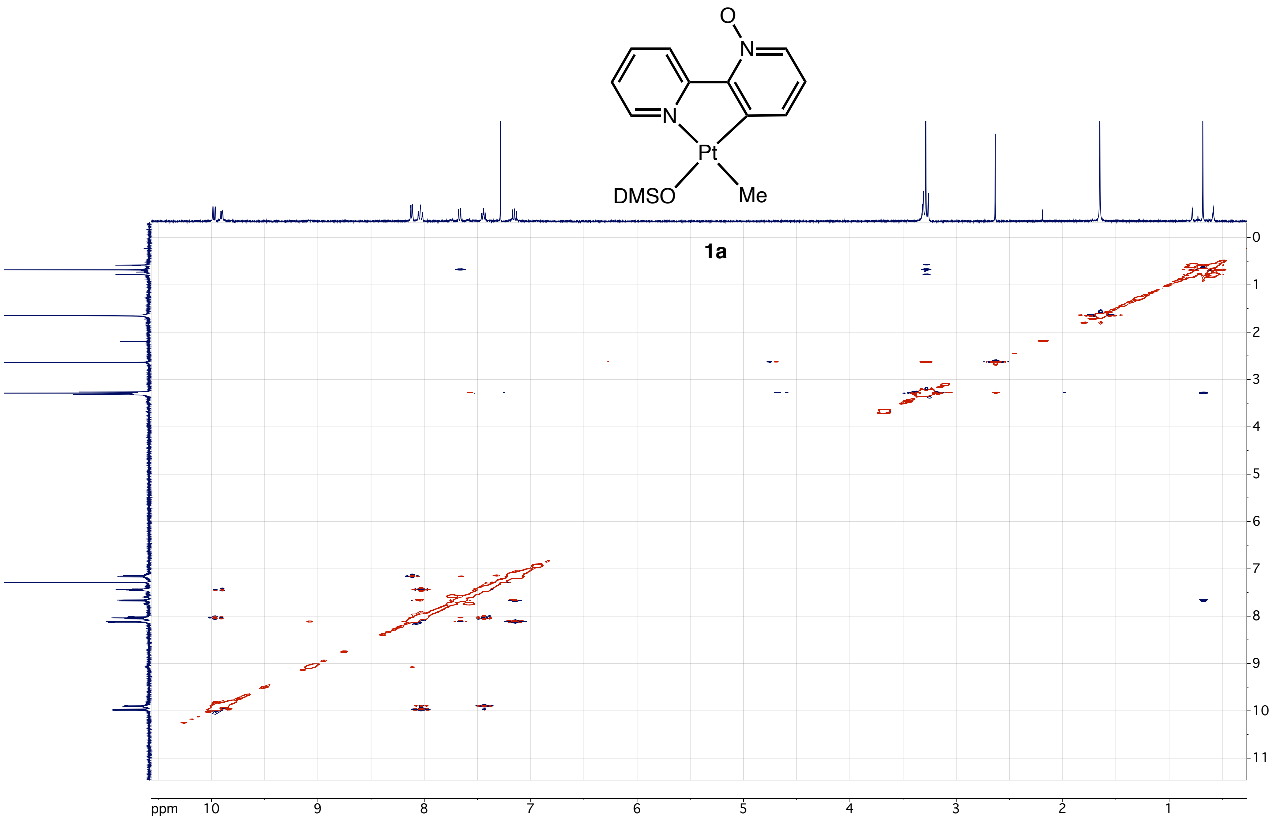

Figure S4. H-H NOESY NMR spectrum ($CDCl_3$) of **1a**

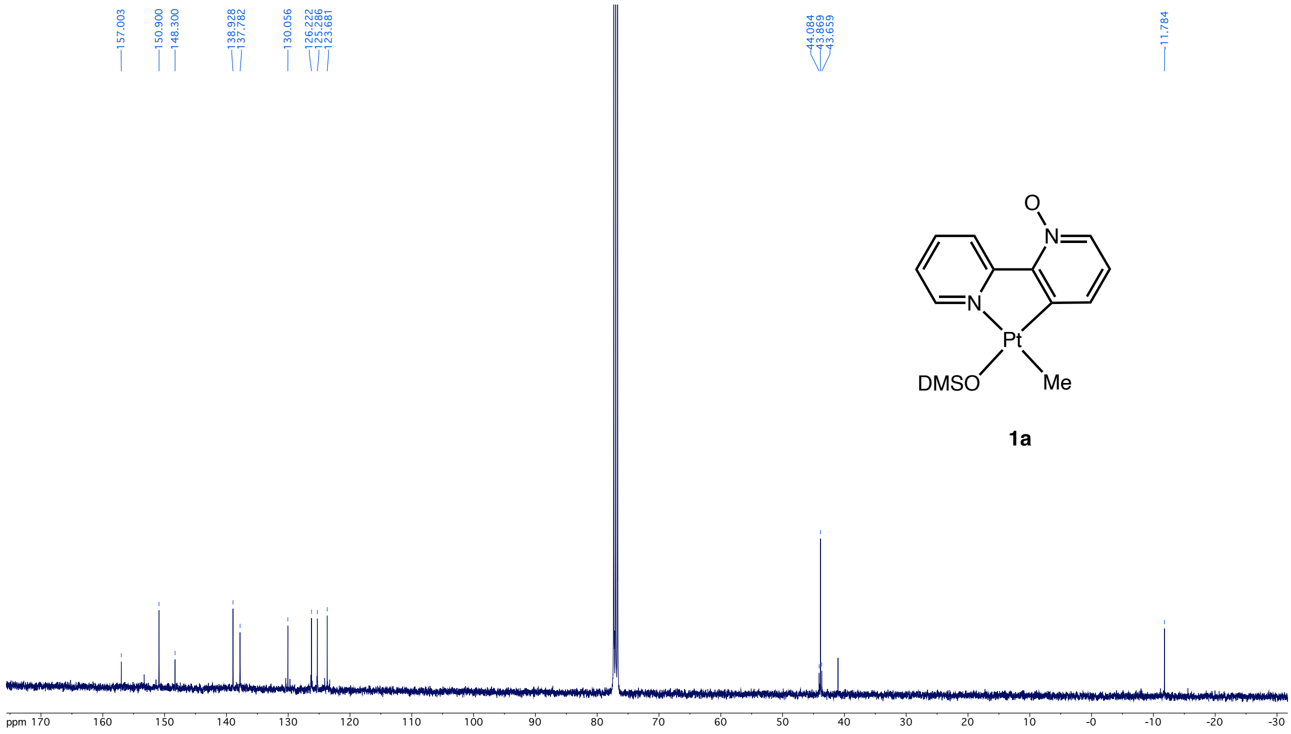

Figure S5. ^{13}C NMR spectrum ($CDCl_3$) of **1a**

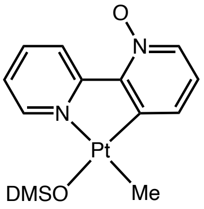

1a

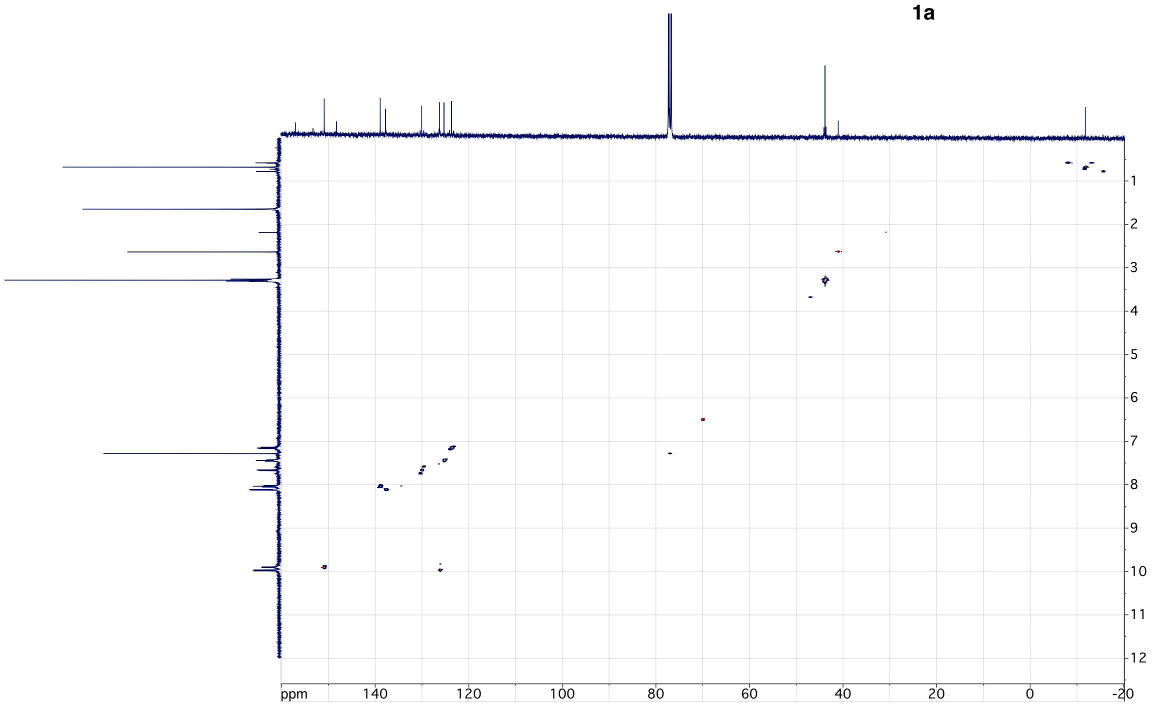

Figure S6. HSQC NMR spectrum ($CDCl_3$) of **1a**

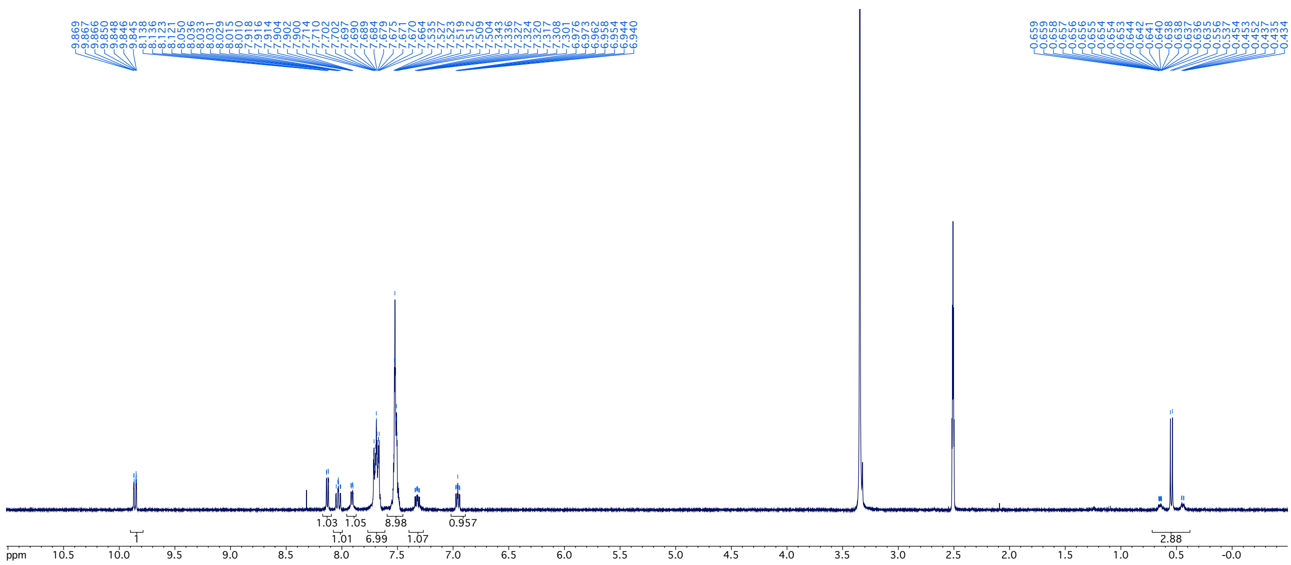

Figure S7. ^{1}H NMR spectrum (DMSO) of **2a**

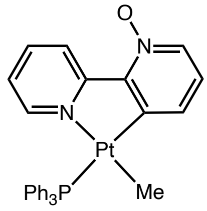

2a

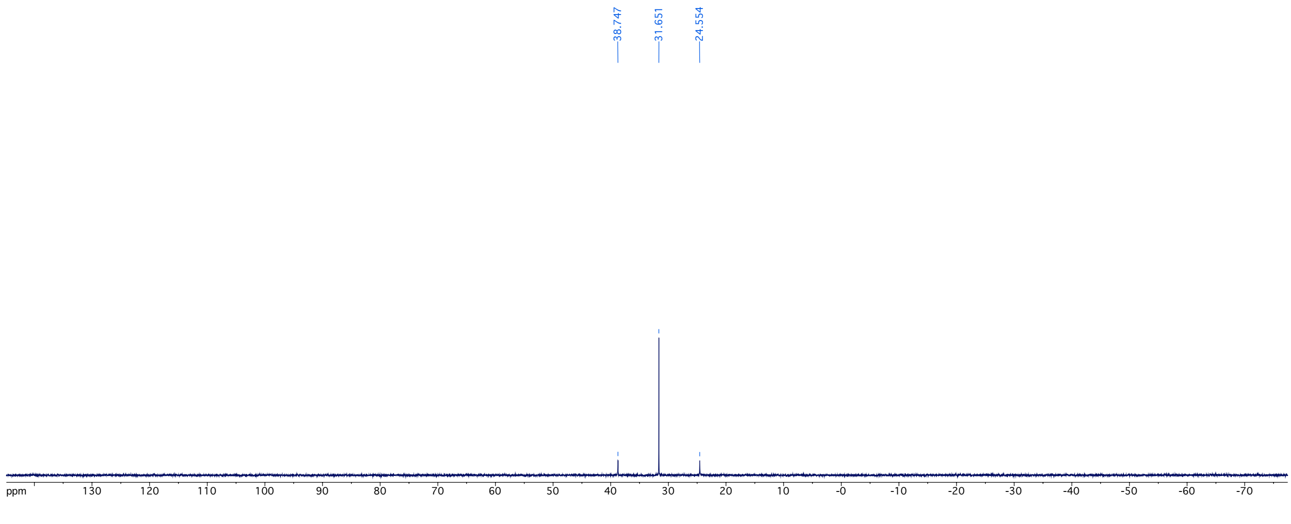

Figure S8. ^{31}P NMR spectrum (DMSO) of **2a**

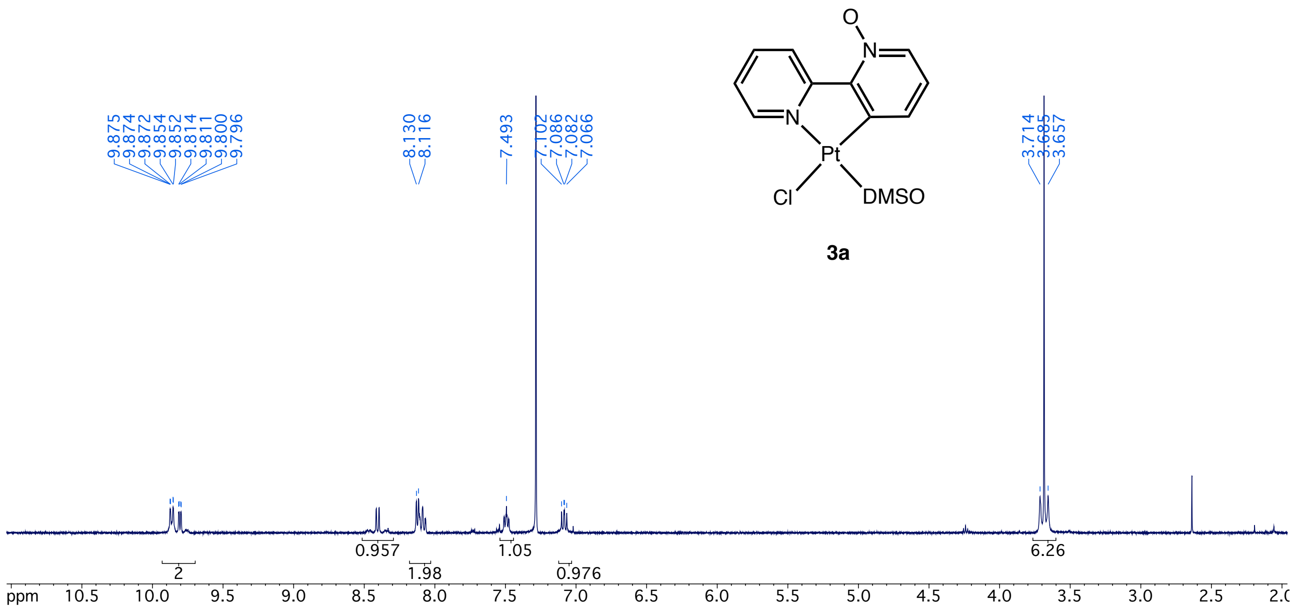

Figure S9. ^{1}H NMR spectrum ($CDCl_3$) of **3a**

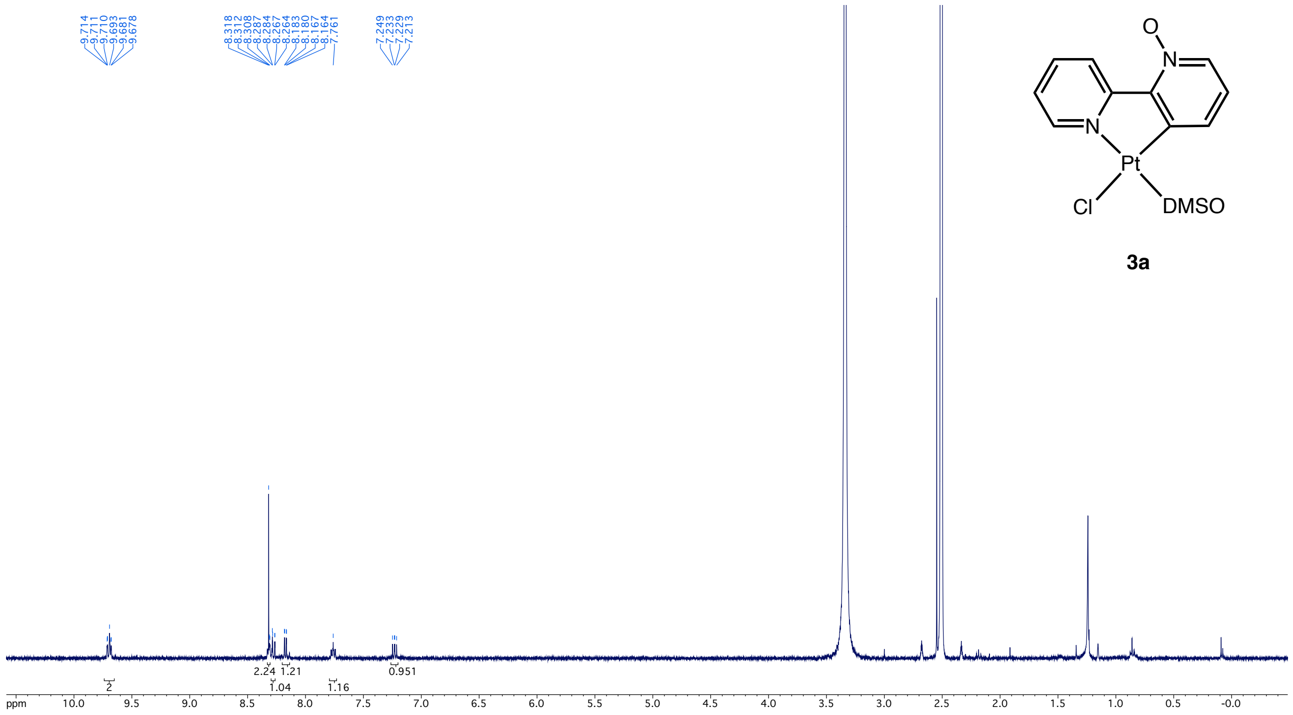

Figure S10 ^{1}H NMR spectrum (DMSO) of **3a**

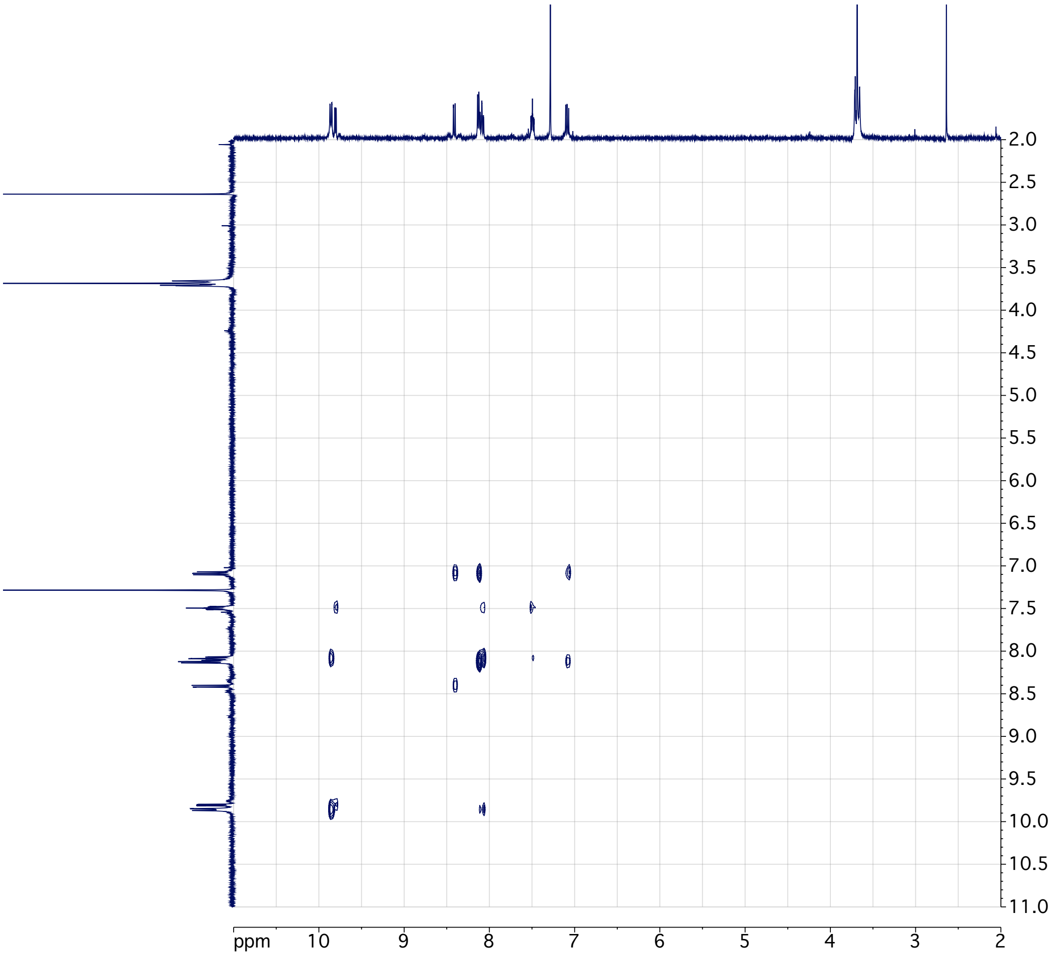

Figure S11. H-H COSY NMR spectrum ($CDCl_3$) of **3a**

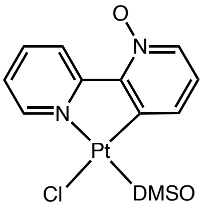

3a

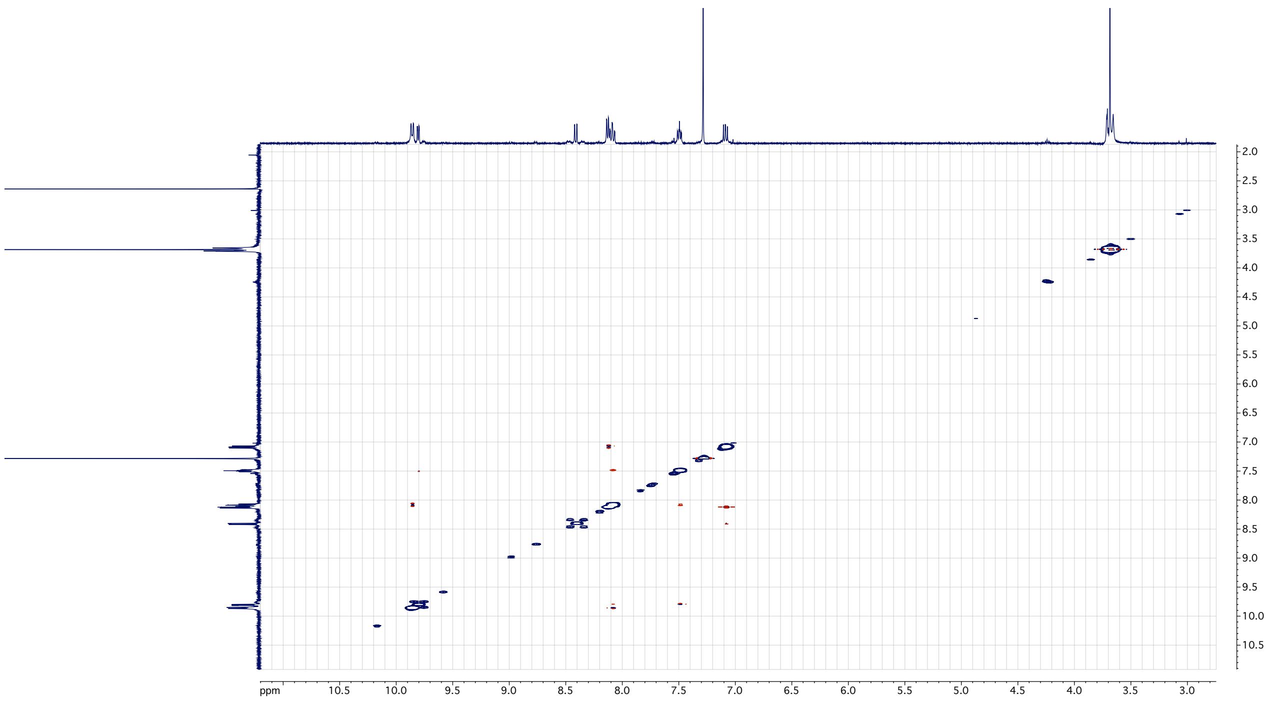

Figure S12. H-H NOESY NMR spectrum ($CDCl_3$) of **3a**

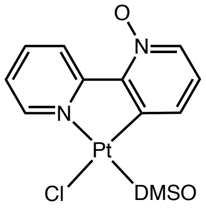

3a

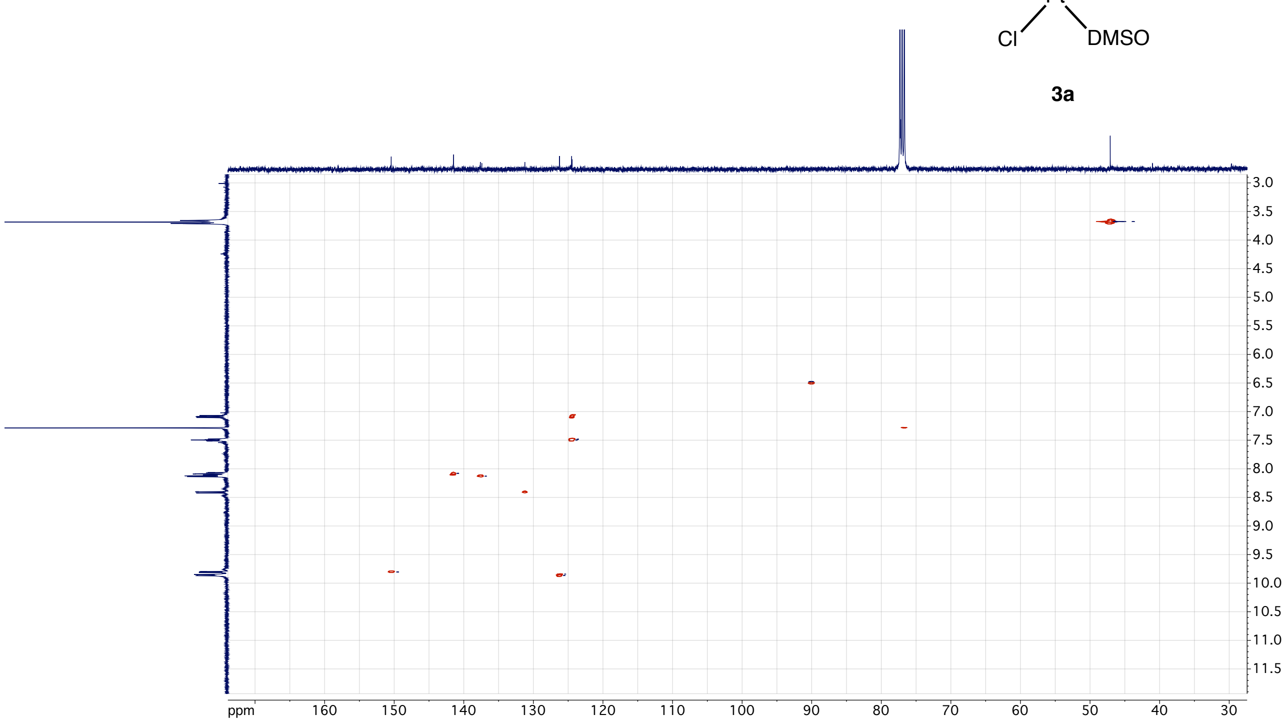

Figure S13. H-C HSQC NMR spectrum ($CDCl_3$) of **3a**

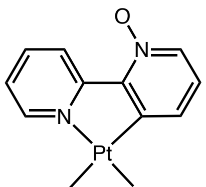

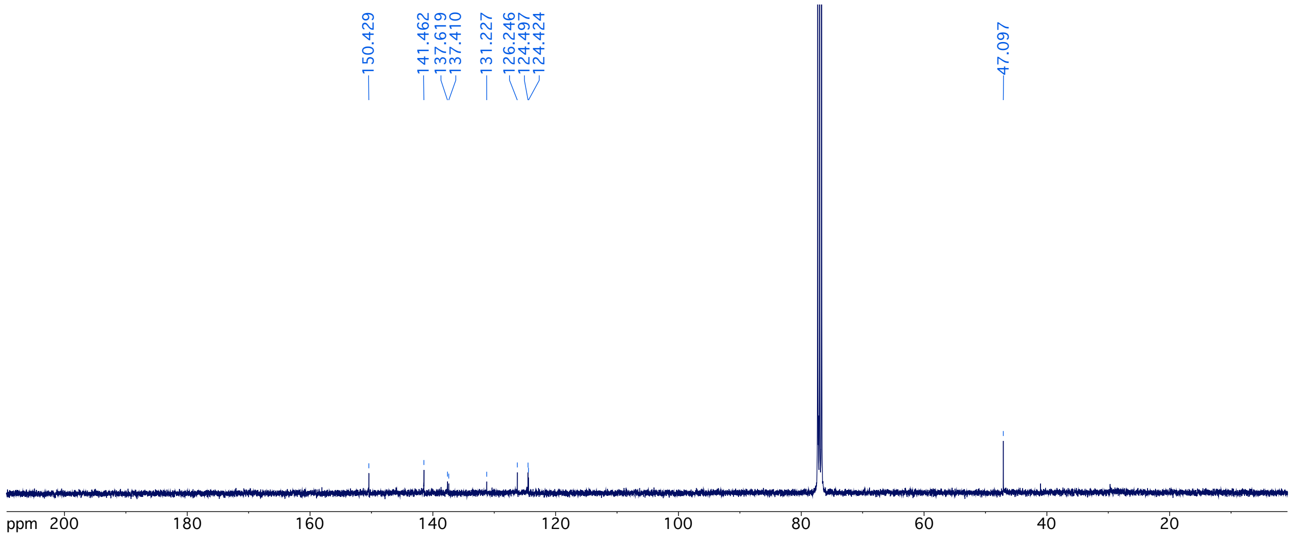

Figure S14. ^{13}C NMR spectrum ($CDCl_3$) of **3a**

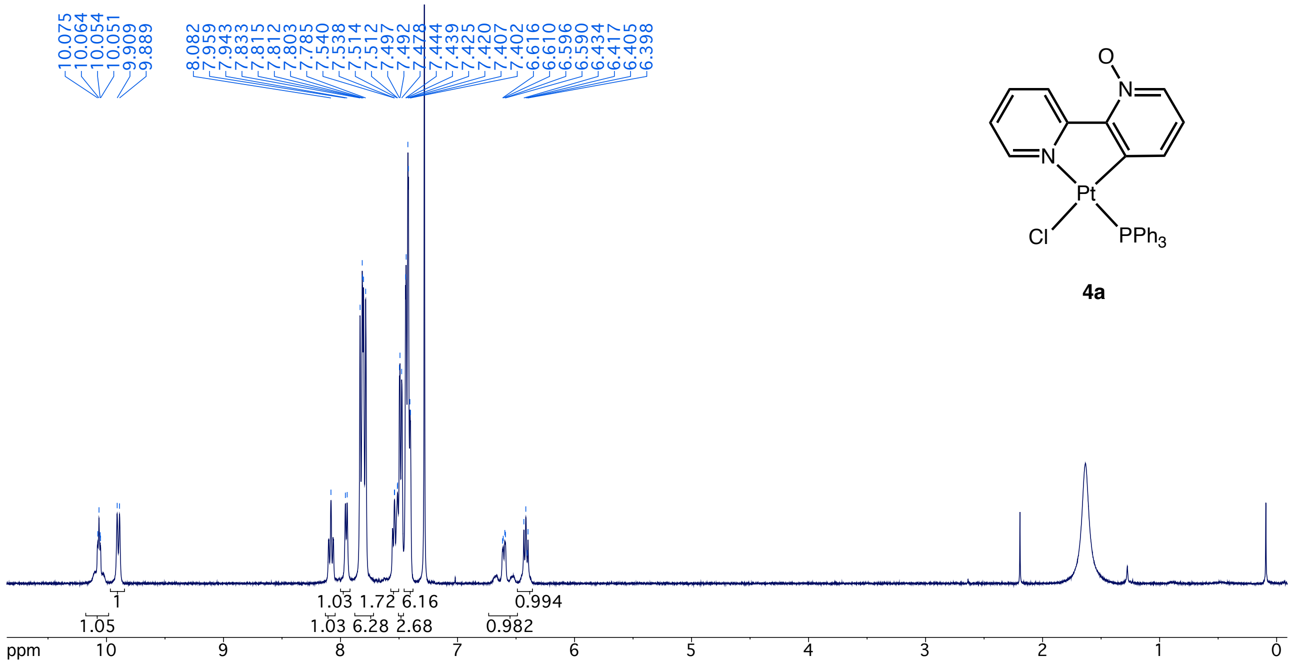

Figure S15. ^{1}H NMR spectrum ($CDCl_3$) of **4a**

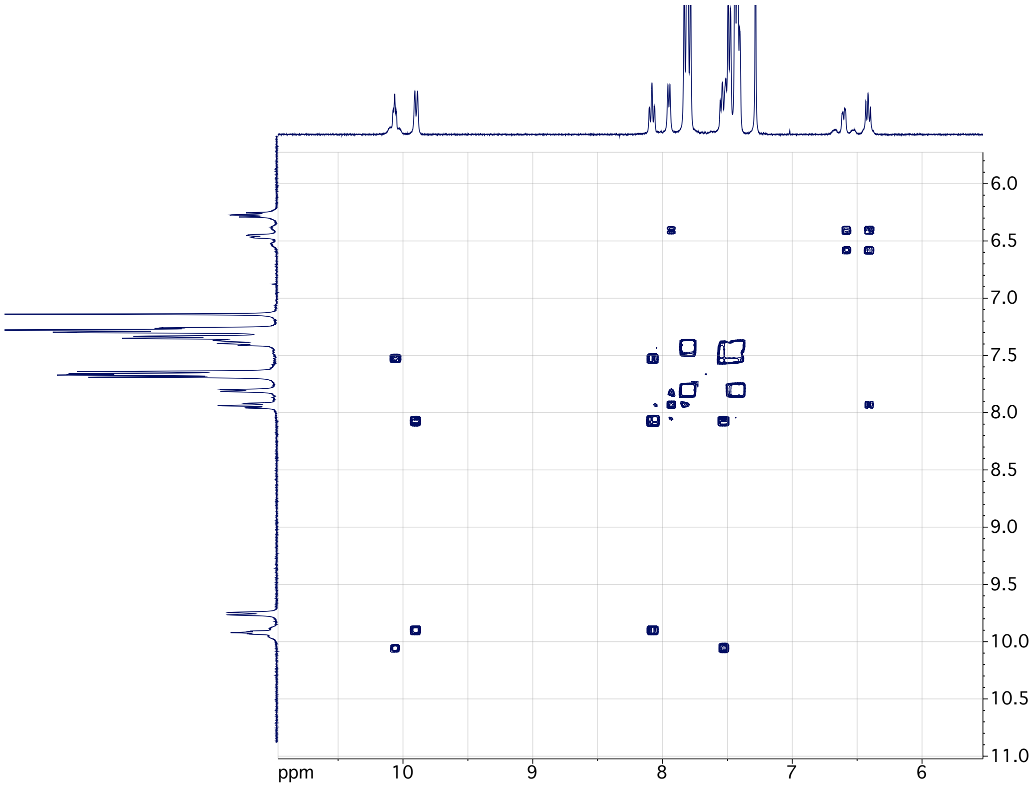

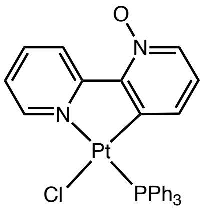

4a

Figure S16. ^{1}H COSY NMR spectrum ($CDCl_3$) of **4a**

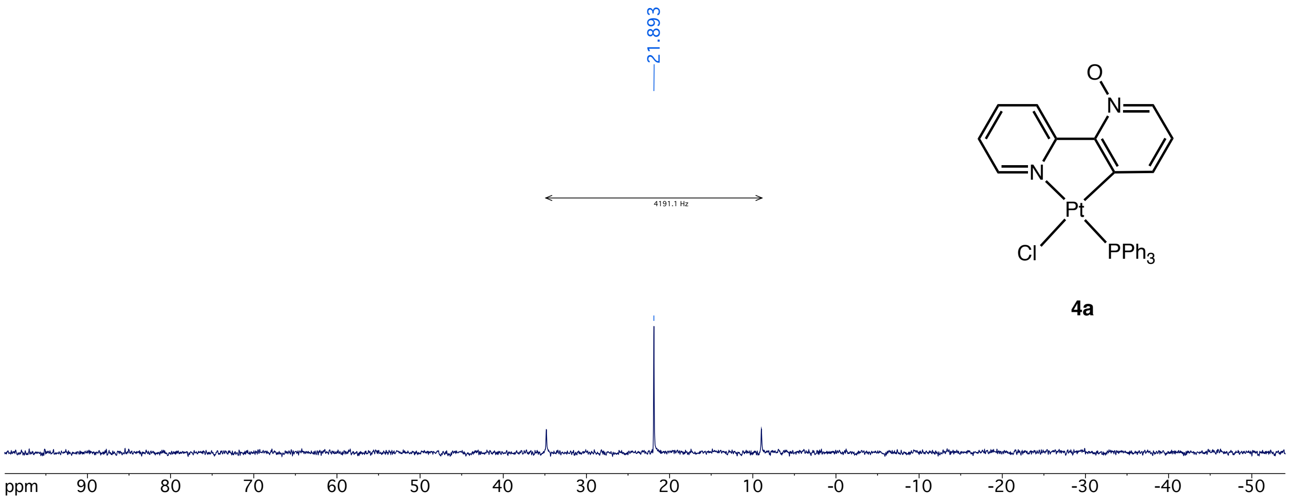

Figure S17. ^{31}P NMR spectrum ($CDCl_3$) of **4a**

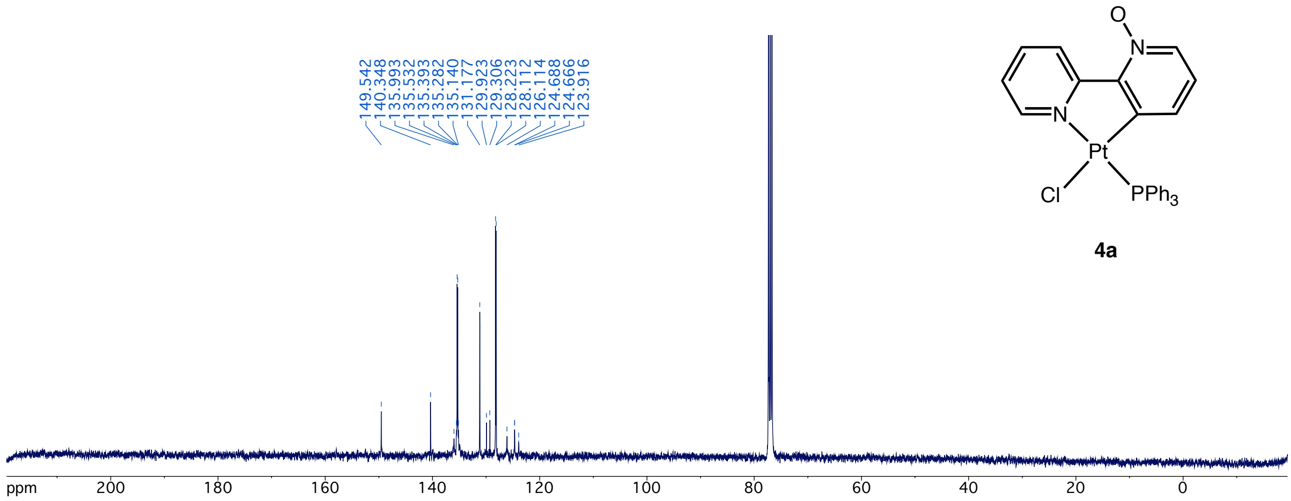

Figure S18. ^{13}C NMR spectrum ($CDCl_3$) of **4a**

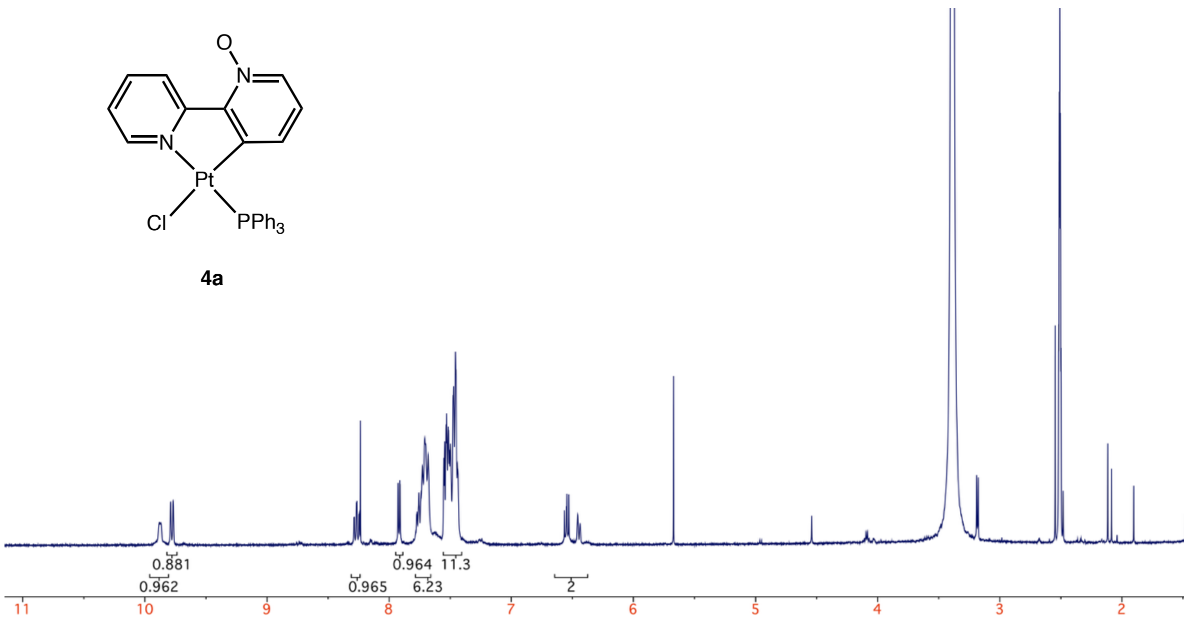

Figure S19. ^{1}H NMR spectrum (DMSO-d$_6$) of **4a**

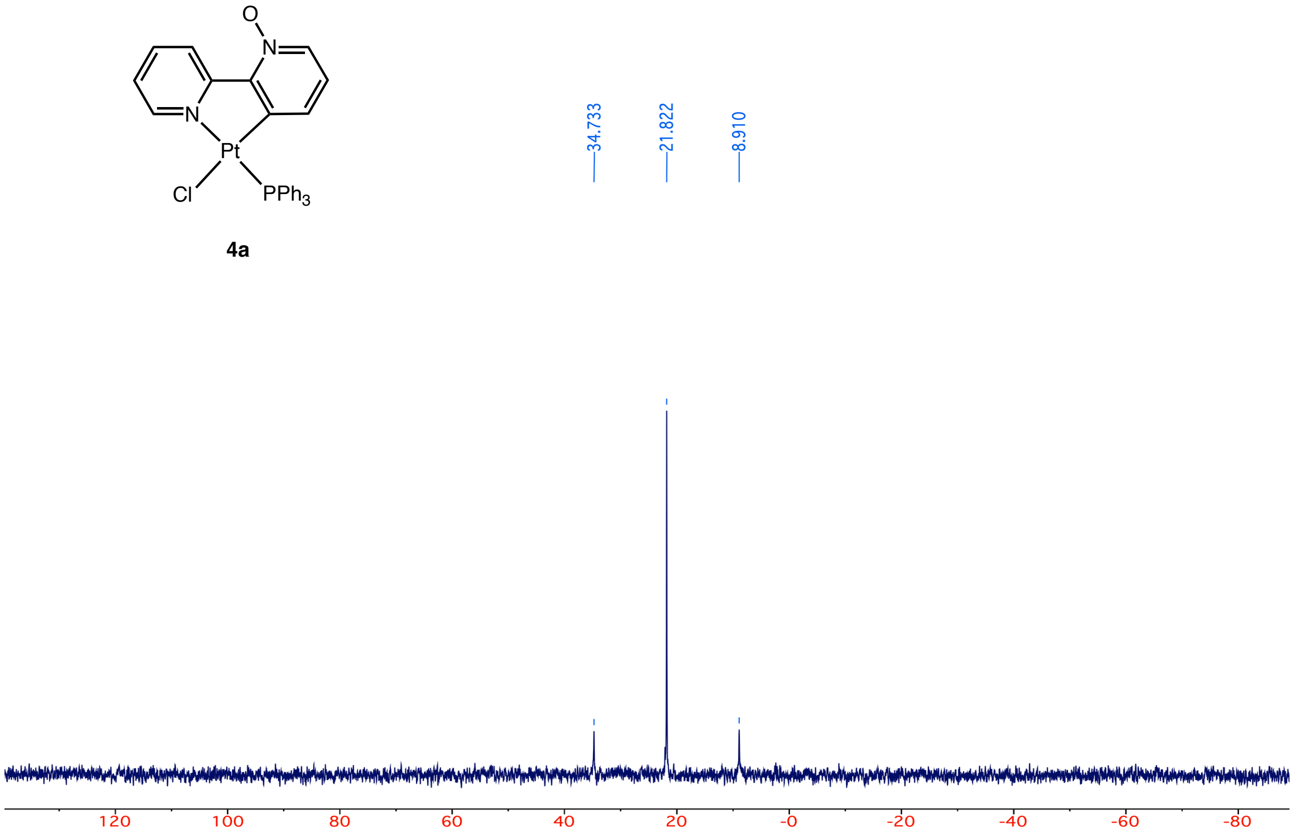

Figure S20. ^{31}P NMR spectrum (DMSO-d$_6$) of **4a**

Table S1. Selected crystallographic data for complex **4a**.

	**4a**
CCDC No.	2512771
formula	$C_{28}H_{22}ClN_2OPPt$
Mr	663.92
Crystal system	monoclinic
Space group	$P2_1/c$
a, Å	14.7007(5)
b, Å	9.5439(3)
c, Å	34.1580(11)
α, deg.	90
β, deg.	90.721(2)
γ, deg.	90
V, Å^3	4792.1(3)
Z	8
$\rho_{calc,}$ g/cm^3	1.841
μ, mm^{-1}	12.805
F(000)	2576
No. reflections (unique)	44832(9973)
S^a	1.31
$R_1(wR_2)$ ($F^2 > 2\sigma(F^2)$)	0.0594, 0.1478
R_{int}	0.036
Min./max diff map e Å^{-3}	–3.44, 2.05

^{a}Conventional $R = \Sigma||Fo| - |Fc||/\Sigma|Fo|$; $Rw = [\Sigma w(Fo^2 - Fc^2)^2/\Sigma w(Fo^2)^2]^{1/2}$; $S = [\Sigma w(Fo^2 - Fc^2)^2/\text{no. data} - \text{no. params})]^{1/2}$ for all data.

Table S2. NMR data for other complexes. Letters **a-n** refers to cyclometalated ligand (see Chart 1)

complex Pt(N^C)(DMSO)Me	N^C ligand	^{1}H DMSO δ ($^{3}J_{Pt-H}$)	^{13}C δ ($^{1}J_{Pt-C}$)	^{1}H δ Me	reference
**1a**	bpy^{NO}	3.29 (18.9)		0.68 (80.4)	This paper
**1b**	bpy	3.27 (18.2)	145.1 (1090)	0.70 (82.0)	[41]
**1c**	phpy	3.21 (17.6)	150.4 (1063)	0.66(83.5)	
**1k**	bpy^{CF3}	3.29 (18.5)	149.5 (1100)	0.73 (83.1)	[47]
**1j**	bpy^{OMe}	3.26 (18.4)	136.8 (1092)		[46]
**1m**	bpy*	3.30 (20.5)		0.78 (81)	[37]
**1g**	bpy^{Me}	3.24 (18.3)		0.69 (82.0)	[43]
**1i**	pyqui	3.29 (18.3)	140.80 (1090)	0.82 (82)	[20]
**1n**	pyqui*	3.33 (20.3)		0.85 (81.3)	[20]
$Pt(N^\wedge C)(PPh_3)Cl$					
**4a**	bpy^{NO}	21.8 (4191)			This paper
**4c**	$phpy^{a}$	23.4(4338)			[48]
**4b**	bpy	23.6 (4285)			[41]
**4i**	pyqui	22.4 (4288)			[20]
**4n**	pyqui*	20.1 (4143)			[20]

Table S3. Comparison between Pt-P and Pt-S bond distances in complexes $[Pt(N^\wedge C)(PPh_3)Me]$ and [Pt(N^C)(DMSO)Me]

CCDC identifier	N^C ligand	Pt-P bond distances	CCDC number	reference
		$[Pt(N^\wedge C)(PPh_3)Me]$		
MANFUF	phpy	2.302(3)	1538745	[49]
MANFUF01	phpy	2.2955(9)	1546693	[38]
ONIWIT	bpy^{NO}	2.298(1)	1485320	[28]
YOVJUQ	bpy	2.2904(7)	963378	[50]
		[Pt(N^C)(DMSO)Me]		
		Pt-S bond distances		
AQARIV	bpy^{OMe}	2.270(3)<br>2.268(3)	1436235	[47] 26/06/26 10:40:00
BEZPEC	phpy	2.2759(9)	195233	[39]
